# Meta-Analysis and Systematic Review of Coagulation Disbalances in COVID-19: 41 Studies and 17,601 Patients

**DOI:** 10.3389/fcvm.2022.794092

**Published:** 2022-03-11

**Authors:** Polina Len, Gaukhar Iskakova, Zarina Sautbayeva, Aigul Kussanova, Ainur T. Tauekelova, Madina M. Sugralimova, Anar S. Dautbaeva, Meruert M. Abdieva, Eugene D. Ponomarev, Alexander Tikhonov, Makhabbat S. Bekbossynova, Natasha S. Barteneva

**Affiliations:** ^1^School of Sciences and Humanities, Nazarbayev University, Nur-Sultan, Kazakhstan; ^2^Core Facilities, Nazarbayev University, Nur-Sultan, Kazakhstan; ^3^National Research Center for Cardiac Surgery, Nur-Sultan, Kazakhstan; ^4^School of Biomedical Sciences, The Chinese University of Hong Kong, Hong Kong, China; ^5^Harvard Medical School, Brigham and Women's Hospital, Boston, MA, United States

**Keywords:** COVID-19, coagulopathy, thrombosis, D-dimers, platelets, fibrinogen, prothrombin time, megakaryocyte

## Abstract

**Introduction:**

Coagulation parameters are important determinants for COVID-19 infection. We conducted meta-analysis to assess the association between early hemostatic parameters and infection severity.

**Methods:**

Electronic search was made for papers that addressed clinical characteristics of COVID-19 patients and disease severity. Results were filtered using exclusion and inclusion criteria and then pooled into a meta-analysis to estimate the standardized mean difference (SMD) with 95% confidence interval (CI) for D-dimers, fibrinogen, prothrombin time, platelet count (PLT), activated partial thromboplastin time. To explore the heterogeneity and robustness of our fundings, sensitivity and subgroup analyses were conducted. Publication bias was assessed with contour-enhanced funnel plots and Egger's test by linear regression. Coagulation parameters data from retrospective cohort study of 451 patients with COVID-19 at National Research Center for Cardiac Surgery were included in meta-analysis of published studies.

**Results:**

Overall, 41 original studies (17,601 patients) on SARS-CoV-2 were included. For the two groups of patients, stratified by severity, we identified that D-dimers, fibrinogen, activated partial thromboplastin time, and prothrombin time were significantly higher in the severe group [SMD 0.6985 with 95%CI (0.5155; 0.8815); SMD 0.661 with 95%CI (0.3387; 0.9833); SMD 0.2683 with 95%CI (0.1357; 0.4009); SMD 0.284 with 95%CI (0.1472; 0.4208)]. In contrast, PLT was significantly lower in patients with more severe cases of COVID-19 [SMD −0.1684 with 95%CI (−0.2826; −0.0542)]. Neither the analysis by the leave-one-out method nor the influence diagnostic have identified studies that solely cause significant change in the effect size estimates. Subgroup analysis showed no significant difference between articles originated from different countries but revealed that severity assessment criteria might have influence over estimated effect sizes for platelets and D-dimers. Contour-enhanced funnel plots and the Egger's test for D-dimers and fibrinogen revealed significant asymmetry that might be a sign of publication bias.

**Conclusions:**

The hemostatic laboratory parameters, with exception of platelets, are significantly elevated in patients with severe COVID-19. The two variables with strongest association to disease severity were D-dimers and fibrinogen levels. Future research should aim outside conventional coagulation tests and include analysis of clotting formation and platelet/platelet progenitors characteristics.

## Introduction

In <2 years of the outbreak, the COVID-19 pandemic took almost five million lives (1). SARS-CoV-2 is the seventh member of the large coronavirus family capable of inducing human disease (2). Viral respiratory infections, including severe respiratory acute syndrome coronavirus (SARS-CoV), Middle East respiratory syndrome coronavirus (MERS-CoV), SARS-CoV-2, may induce coagulopathy and lead to intravascular thrombi and deposition of fibrinogen (3–7).

The coagulation system is activated and dysregulated during COVID-19 infection; however, characteristics of COVID-19 associated coagulopathy are different from other coagulation disorders (8). Evidence of abnormal COVID-19-associated coagulation parameters has already appeared in early publications from Wuhan (9–12) and was supported by further publications worldwide [rev. (8, 13)]. The reported hemostatic abnormalities with COVID-19 infection include increased D-dimers (DD) levels (14, 15), fibrinogen levels (16, 17), changes in the quantities and levels of activation of platelets (14, 18–20), elevated von Willebrand factor (VWF) [(21, 22), rev. by Becker et al. (23)] and other coagulation parameters. Persistently elevated risk of thromboembolic events, even with the initiation of prophylactic anti-coagulation, suggest the presence of hypofibrinolysis, in addition to detected hypercoagulability in COVID-19 (24, 25). It is also consistent with the results of thromboelastography (TEG) reported by Wright et al. (26).

There is a need for early detection of elevated coagulation biomarkers to optimize risk stratification of patients with COVID-19. Importantly, multiple findings suggest that thromboembolic complications should be considered as a cause of clinical deterioration in severe COVID-19 infection and potentially beyond hospital discharge (27–29). However, due to the limitations of early pandemic research with small study sizes and highly heterogeneous datasets, it is still debated. The aim of the current study was to evaluate the validity of stratification based on early coagulation parameters provided by different clinical studies worldwide. We also aimed to use subgroup analysis to explore whether differences in patient subpopulations partly explained heterogeneity in results. The coagulation parameters of our own cohort were included in a systematic meta-analysis of published studies. Furthermore, we also discuss a complex mechanism of hemostatic dysregulation that fuels the coagulation cycle.

## Materials and Methods

In this study, we aimed to identify the relationship between coagulation biomarkers taken at admission and severity of COVID-19 in adult (above 18) patients by estimating the effect size of five laboratory coagulation tests: D-dimers (DD), platelet count (PLT), fibrinogen (FIB), activated partial thromboplastin time (APTT), and prothrombin time (PT).

### Search Strategy, Exclusion, and Inclusion Criteria

To broaden our search, we included Ovid, PubMed, Web of Sciences, and Google Scholar in the study for the period ending September 30th, 2021. They were thoroughly scanned using the following keywords: SARS-CoV-2, COVID-19, coagulation, severity, characteristics, features, D-dimers, platelets, fibrinogen, cohort, observational, retrospective. First, satisfying search results were exported to an Excel table, and duplicates were eliminated. Second, we conducted a preliminary review of retrieved articles' abstracts. Papers that focused on pregnant women, children, or specific age groups were avoided. Similarly, works that focused on the effect of medical treatment or patients with particular commodities were not included in the meta-analysis. We also excluded studies that compared mortality among groups of COVID-19 patients. While diagnostic and classification of COVID-19 severity follow official guidelines, the differentiation between whether COVID-19 caused deaths might be ambiguous. In order to avoid such bias, we only considered studies that grouped patients according to the severity of their symptoms. Then, each publication was checked according to the inclusion criteria: sample size, severity assessment criteria, presence of at least one coagulation marker of interest expressed as a continuous variable. To be included, a study should have had more than 15 patients and divided them according to the disease severity—WHO guidelines, intensive care unit (ICU) admission, disease aggravation, or need for oxygen therapy. Next, a list of biomarkers should have included at least some of the coagulation parameters: PLT, DD, FIB, APTT, PT, thrombin time (TT), Activated thrombin (AT), Factor VIII (FVIII), and VWF. Finally, the quality of selected articles was assessed with the NIH Quality Assessment Tool for Observational Cohort and Cross-Sectional Studies (30) (Supplementary Table 1). Questions 5, 6, 12, 13 were not applicable to selected studies due to their observational/retrospective nature; Question 8 was not applicable to studies with dichotomous exposure values, like ICU admission and need for oxygen therapy. Hence, studies with a quality score of <5 were excluded from the analysis. Search and quality check were conducted independently by two authors. Disagreements were resolved during a joint discussion with a third author.

### Data Extraction

The primary goal was to retrieve data from each article about two groups of patients differing in disease severity by: group size, stratification criteria, and results of the laboratory tests. Apart from these, we kept a record of the country, hospital, and admission time to identify and exclude duplicate patients.

### Cohort From the National Research Center for Cardiac Surgery

#### Participants and Data Collection

From a large cohort of 560 patients consecutively admitted from June 2020 to August 2021 due to coronavirus infection to the cardiology department of the National Research Center for Cardiac Surgery, Nur-Sultan, Kazakhstan, we selected 451 patients with a confirmed diagnosis of COVID-19, aged ≥18 years, and those with the onset of the disease ≤ 21 days, all of whom were hospitalized for at least 24 h with COVID-19. All demographic, clinical, laboratory data were extracted from the electronic records of the NRCSC. Laboratory tests included PLT, PT, international normalized ratio, APTT, FIB, DD. Blood samples were collected at admission by the clinical team. Clotting tests were performed according to standard methods. For coagulation tests (PT, international normalized ratio, APTT, FIB), we used AVATUBE® with sodium citrate, and it was measured on Sysmex CS-2500 (Sysmex, Japan). Blood for DD was collected in AVATUBE® with sodium citrate, and D-dimers were quantified on Cobas® 6000. PLT counts were measured on Sysmex XS-500 (Sysmex, Japan) with blood collection performed in AVATUBE® with K2 EDTA. The confirmation of SARS-CoV-2 infection was done by real-time quantitative reverse-transcription polymerase chain reaction (RT-PCR) assay on nose/throat swab or sputum samples using CFX96® Real-Time System (Bio-Rad Laboratories, Inc., USA).

#### Clinical Assessment

The COVID-19 population was initially divided into four groups according to disease severity following local guidelines “Diagnosis and treatment of Covid-19 in Adults” (http://www.rcrz.kz/index.php/ru/2017-03-12-10-51-13/klinicheskie-protokoly) (Supplementary Data 1). The study protocol was approved by the medical ethics committee of the National Research Center for Cardiac Surgery, Nur-Sultan, Kazakhstan waiving the need for written informed consent due to the retrospective design.

### Data Analysis

#### Data Transformation

All data analysis was performed in the RStudio 1.4.1717 with R version 4.1.0 (Integrated Development Environment for R. RStudio, PBC, USA). In most articles, results were reported as the first, second (median), and third quartiles, reflecting that original data might be abnormally distributed. Since the authors of the primary studies do not report the underlying distributions, and there are no established methods to analyze the difference of medians, we performed data transformation according to the method described by McGrath et al. (31). The workflow takes advantage of the formulas proposed by Luo et al. (32) and Wan et al. (33) for estimation of mean and standard deviation, respectively. Both methods assume the input data to be normally distributed, so the package also includes the preceding transformation step by the Box-Cox method. In the scope of this meta-analysis, we compare two groups: more and less severe. Patients classified as severe, critical, admitted to ICU, with observed aggravation of a disease, or in need of oxygen therapy were assigned to the first group. The second group included mild, moderate, outpatients, general ward patients, recovered and stable patients (Supplementary Table 2). If, for example, a primary study had more than two groups—mild and moderate, severe and critical—then mild patients were combined with moderate, and severe were combined with critical.

#### Meta-Analysis

The “meta” and “metafor” packages were used for the meta-analysis. Heterogeneity was assessed using the *I*^2^, tau-squared, and prediction intervals. Following the recommendations of Veroniki et al. (34), Paule-Mandel (PM) tau-squared estimator and Q-Profile (QP) tau-squared confidence interval estimator were used in this meta-analysis. To account for different measurement scales, the standardized mean difference (SMD), or Hedges g was estimated. Before pooling point estimates, each study was assigned a weight based on inverse variance approach—smaller variance gives higher precision, thus yielding greater weight for a study. For datasets with high heterogeneity, the Knapp-Hartung adjusted random-effects model is implemented. In case of low heterogeneity, the fixed-effects model is applied.

#### Sensitivity and Subgroup Analysis

We also performed several sensitivity analyses using the “dmetar” package. First, we recalculated the effect size using the leave-one-out method. Second, basic outliers, studies whose confidence intervals do not overlap with the pooled effect confidence interval, were removed. Third, we ran the diagnostic for influential cases with Graphic Display of Study Heterogeneity (GOSH) plots and excluded them from the meta-analysis. To further explore the heterogeneity of the data, we conducted subgroup analyses: based on grouping criteria and location.

### Publication Bias

We used the counter-enhanced Funnel plots and Egger's test by linear regression to assess the possibility for publication bias in selected studies.

## Results

### Results of the Web Search

**Figure 1:**
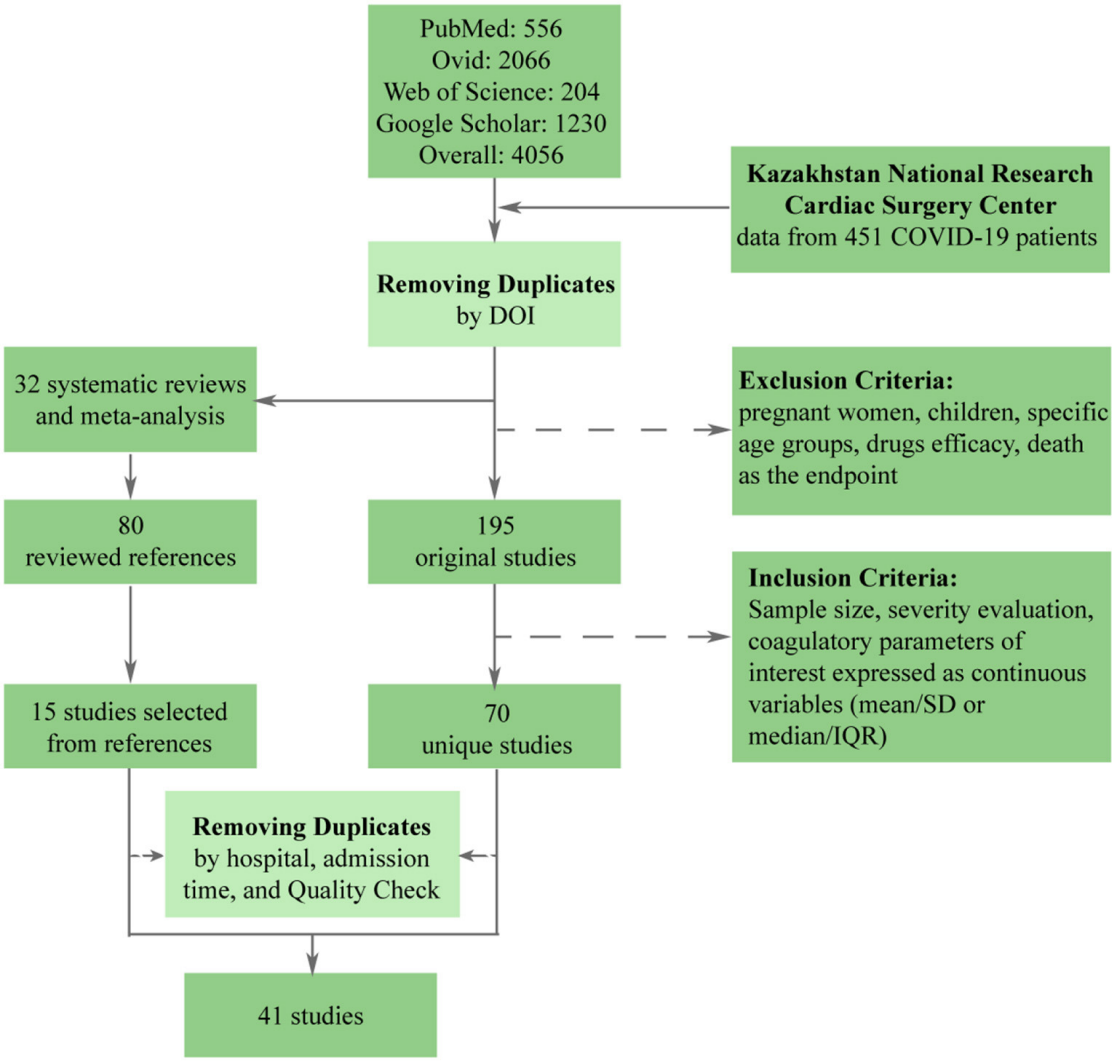
Flow diagram illustrating the process of data collection.

The stepwise process of selection is depicted in Figure 1. Initially, there were 4,056 papers that matched search parameters. After the removal of duplicates and studies that did not meet the exclusion criteria, only 195 unique studies remained. A more thorough review based on the inclusion criteria has proven 125 studies to be unfitting. In addition to 70 that were left, 15 studies were extracted from the references lists of previously published meta-analyses and systematic reviews. Finally, we performed a quality check and removed duplicate patients, which resulted in 40 studies being included in the meta-analysis. Besides original articles, we have included data from the National Research Center for Cardiac Surgery located in Nur-Sultan, Kazakhstan. This data set included data from 451 patients with acute COVID-19.

### Description of Selected Studies

**Figure 2:**
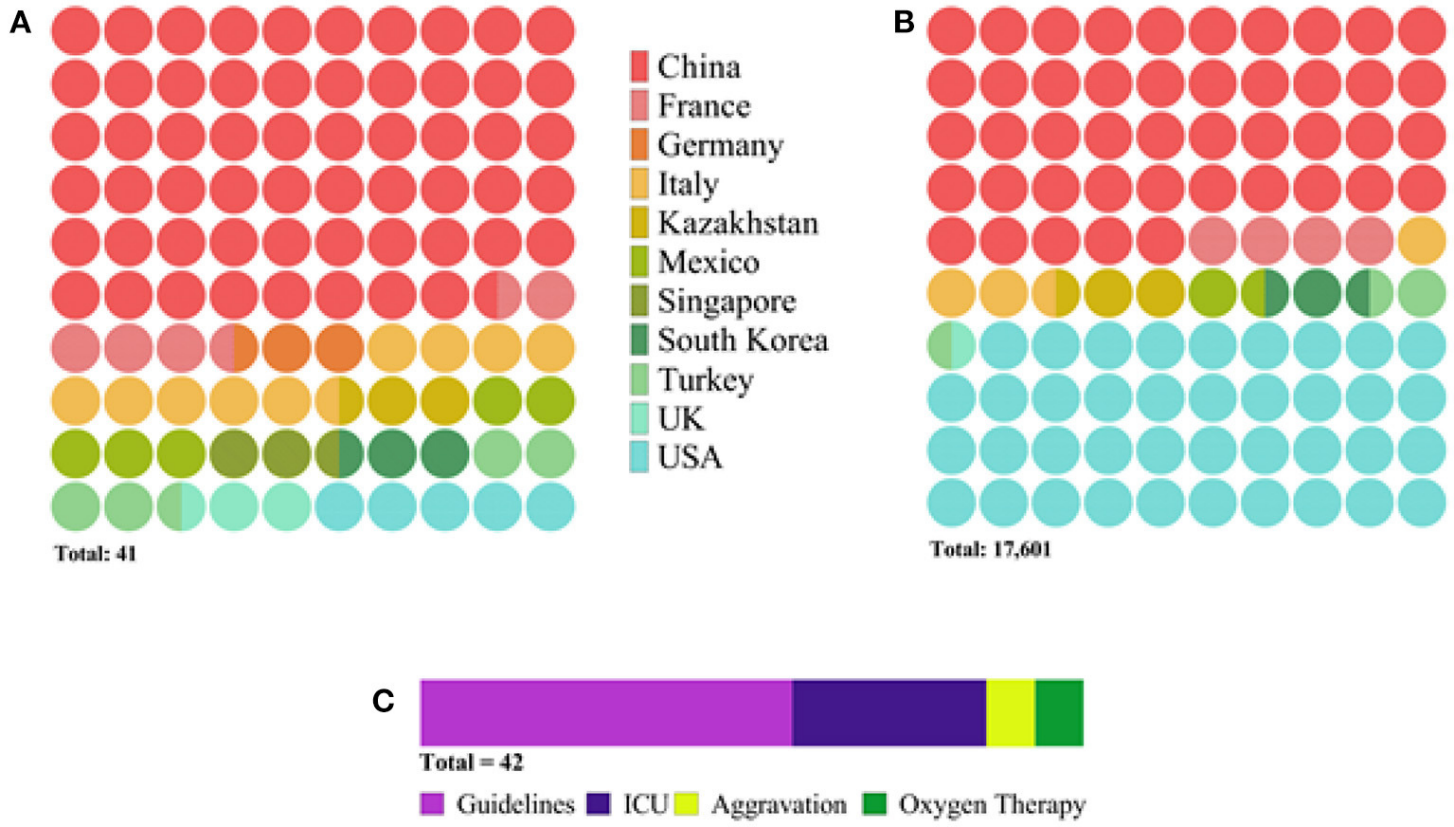
Diagram depicting characteristics of selected studies: **(A)** number of publications per country, **(B)** number of patients per country, and **(C)** number of publications per severity assessment criterion. The latter **(C)** indicates the total number of research articles to be 42 instead of 41 because one study reported two populations of patients admitted to the hospital at different periods.

Overall, this meta-analysis comprises 17,601 patients from 40 research papers and the NRCSC. Of the total, 24 studies originated from China, while the rest included data from France, Germany, Italy, Mexico, Singapore, South Korea, Turkey, the UK, and the USA. Most of the articles reported the division of patients according to the WHO guidelines. Out of the rest, 12 articles made groups based on the ICU admission, three focused on disease aggravation, and three on the need for oxygen therapy. This information is depicted in Figure 2 in more detail.

### Results of Meta-Analysis

#### Pooled Effect Sizes

**Figure 3:**
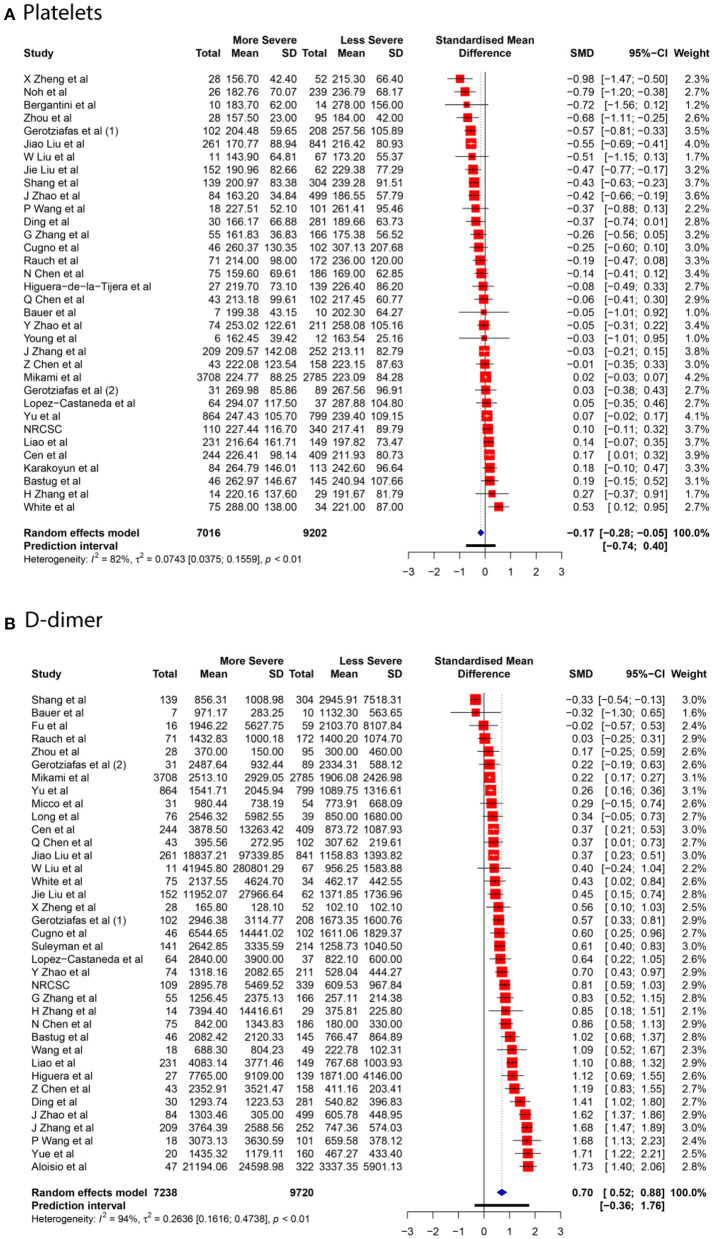
Forest plot of association between COVID-19 severity and PLT **(A)**, DD **(B)**.

**Figure 4:**
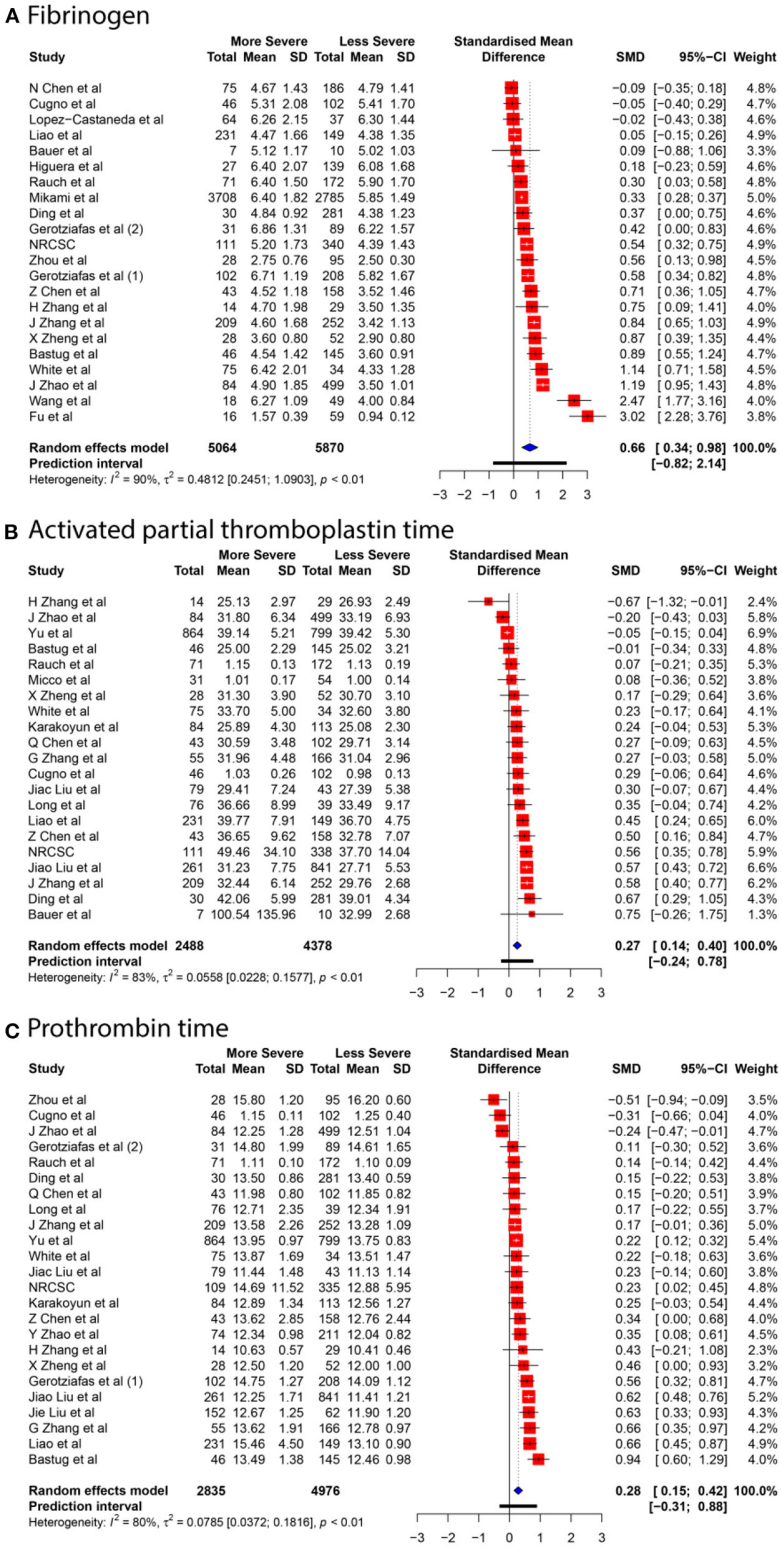
Forest plots of the association between COVID-19 severity and **(A)** FIB, **(B)** APTT, **(C)** PT.

We collected data for five coagulation parameters: PLT, DD, FIB, APTT, and PT. In all cases, the heterogeneity was extremely high (*I*^2^ > 80%), so the Knapp-Hartung adjusted random-effects model was applied in all parts of the meta-analysis. DD, FIB, APTT, and PT were significantly higher in more severe cases [SMD 0.6985 with 95% confidence interval (CI) (0.5155; 0.8815); SMD 0.661 with 95% CI (0.3387; 0.9833); SMD 0.2683 with 95% CI (0.1357; 0.4009); SMD 0.284 with 95% CI (0.1472; 0.4208)]. In contrast, PLT was significantly lower in patients with severe cases of COVID-19 [SMD −0.1684 with 95% CI (−0.2826; −0.0542)]. Forest plots in Figures 3, 4 summarize the results of meta-analyses. Point estimates are represented by red squares, and confidence intervals are shown by corresponding horizontal bars. The sizes of red squares are proportional to weights of respective studies, which are adjusted random-effects weights: reciprocal of the sum of squares of study's standard error and the variance of the distribution of true effect sizes. Pooled effect size and its confidence intervals are shown by blue diamond at the bottom. Detailed output of the meta-analysis is given in Table 1.

**Table 1:** Summary of the effect sizes for all coagulation parameters.

	**Effect size**				**Heterogeneity**		
**Parameter**	**Pooled SMD**	**95% CI**	***t***	***p*-value**	**I ^**∧**^2 (%)**	**Tau-Squared**	***p*-value**
PLT	−0.1684	[−0.2826; −0.0542]	−3.00	0.0051	82.2	0.0743	<0.0001
DD	0.6985	[0.5155; 0.8815]	7.74	<0.0001	94.2	0.2636	<0.0001
FIB	0.6610	[0.3387; 0.9833]	4.27	0.0003	90.4	0.4812	<0.0001
APTT	0.2683	[0.1357; 0.4009]	4.22	0.0004	82.8	0.0558	<0.0001
PT	0.2840	[0.1472; 0.4208]	4.30	0.0003	80.0	0.0785	<0.0001

#### Sensitivity and Subgroup Analysis

Neither the analysis by the leave-one-out method nor the influence diagnostic has identified studies that solely cause a significant change in the effect size estimates. Removal of several outliers based on the pooled and individual confidence intervals allowed significant decrease in heterogeneity, yet it did not affect the interpretation of the results. Adjusted effect sizes and heterogeneity measurements are summarized in Table 2. All influence diagnostic plots, such as Baujat and leave-one-out plots, Cook's distance, Covariance Ratio, etc., are provided in the Supplementary Figures 1–16 along with resulting Forest plots.

**Table 2:** Summary results of the sensitivity analysis.

		**Effect size**				**Heterogeneity**		
**Coagulation parameter**		**Pooled SMD**	**95% CI**	***t***	***p*-value**	***I^**2**^* (%)**	**Tau–Squared**	***Q*-test *p*-value**
PLT	Original	−0.1684	[−0.2826; −0.0542]	−3.00	0.0051	82.2	0.0743	<0.0001
	Outliers removed	−0.1416	[−0.2419; −0.0412]	-2.90	0.0076	56.3	0.0287	0.0002
	Inf. stud. removed	–	–	–	–	–	–	–
DD	Original	0.6985	[0.5155; 0.8815]	7.74	<0.0001	94.2	0.2636	<0.0001
	Outliers removed	0.6210	[0.4860; 0.7560]	9.47	<0.0001	70.2	0.0730	<0.0001
	Inf. stud. removed	–	–	–	–	–	–	–
FIB	Original	0.6610	[0.3387; 0.9833]	4.27	0.0003	90.4	0.4812	<0.0001
	Outliers removed	0.5889	[0.4141; 0.7638]	7.14	<0.0001	85.0	0.0801	<0.0001
	Inf. stud. removed	0.4855	[0.3030; 0.6679]	5.57	<0.0001	86.1	0.1175	<0.0001
APTT	Original	0.2683	[0.1357; 0.4009]	4.22	0.0004	82.8	0.0558	<0.0001
	Outliers removed	0.3480	[0.2450; 0.4518]	7.14	<0.0001	38.6	0.0125	0.0532
	Inf. stud. removed	0.3214	[0.2036; 0.4392]	5.73	<0.0001	68.8	0.0326	<0.0001
PT	Original	0.2840	[0.1472; 0.4208]	4.30	0.0003	80.0	0.0785	<0.0001
	Outliers removed	0.2930	[0.2099; 0.3761]	7.44	<0.0001	26.9	0.0064	0.1407
	Inf. stud. removed	0.2830	[0.1336; 0.4325]	3.94	0.0008	81.0	0.0877	<0.0001

**Figure 5:**
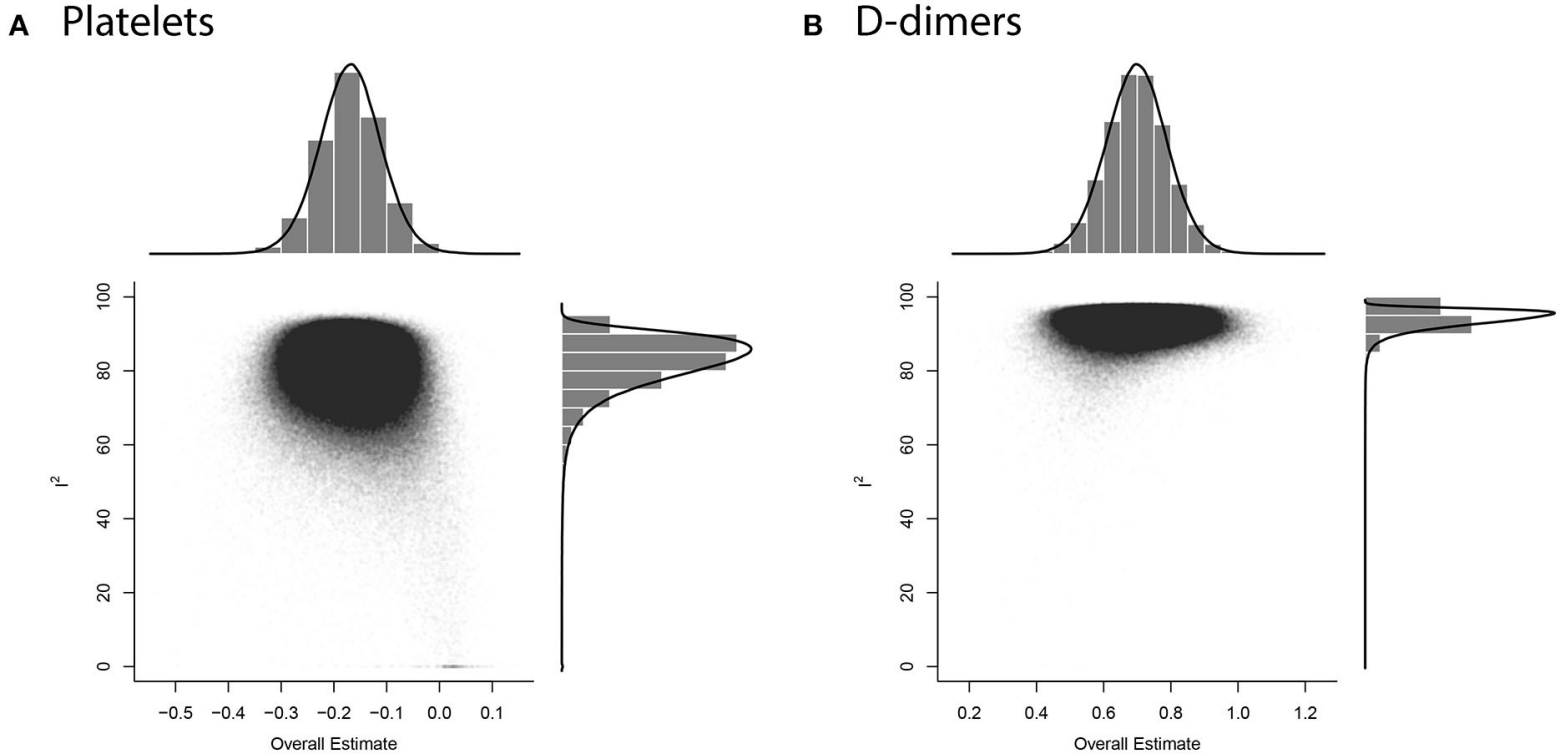
GOSH diagnostic for influential cases in meta-analysis models **(A)** PLT, **(B)** DD.

**Figure 6:**
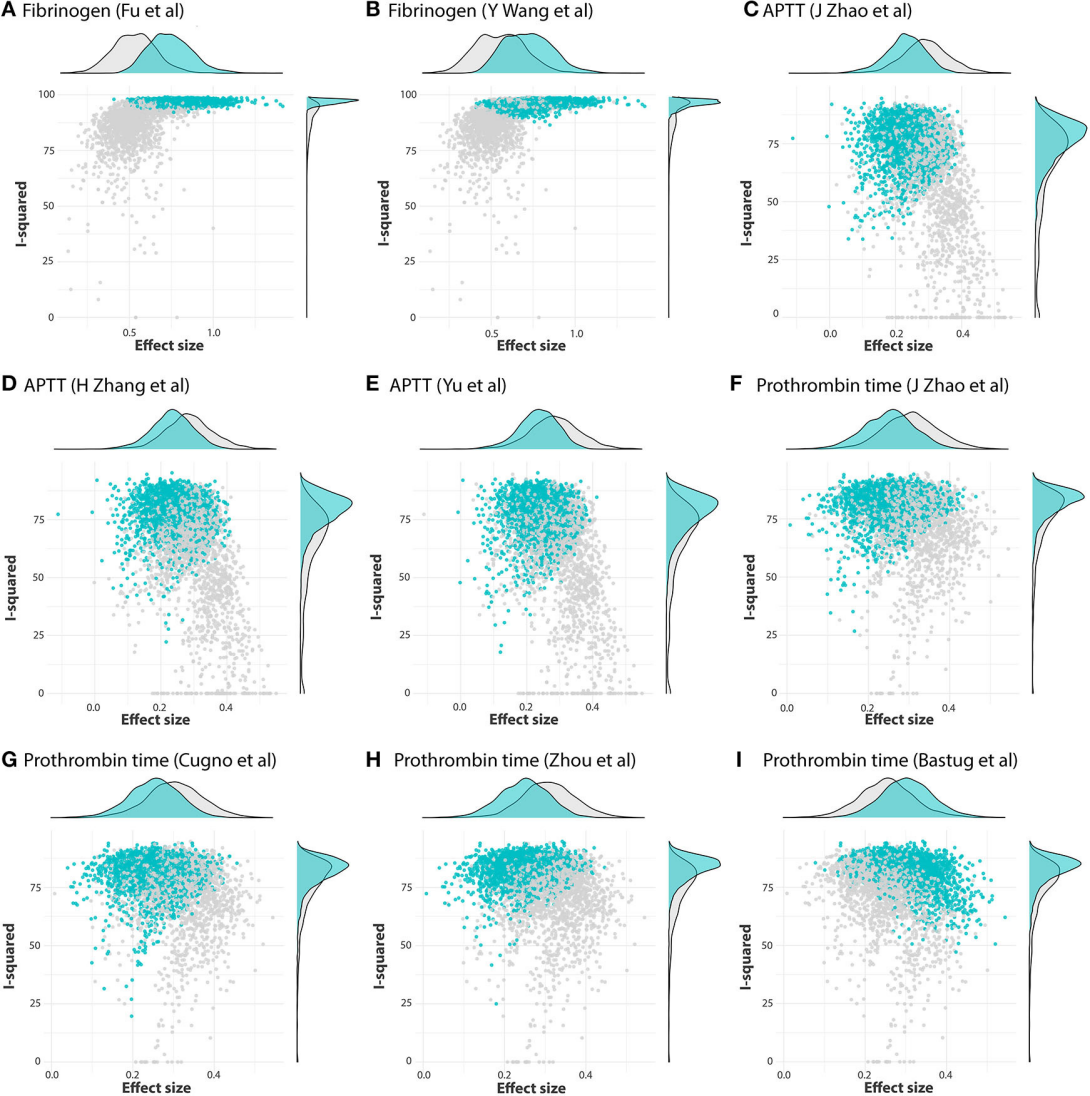
GOSH plots fixed for influential studies in meta-analysis models. **(A,B)** FIB, **(C–E)** APTT, and **(F-I)** PT.

GOSH plots were built for all five models. K-means, DBSCAN, and Gaussian Mixture Model were implemented as clustering methods. This diagnostic was not applied on PLT and DD since they only have one cluster each (Figure 5); for FIB, APTT, and PT, on the other hand, we constructed the plots and identified several possible influential cases. Original GOSH plots for these parameters are given at Supplementary Figures. For fibrinogen, models that include studies by Fu et al. (35) and Wang et al. (12) tend to show higher heterogeneity and greater effect size (Figures 6A,B). For activated partial thromboplastin time, the GOSH plot demonstrated that studies by Zhao et al. (36), Zhang et al. (37), and Yu et al. (38) increase the model's heterogeneity and slightly pull the effect size to the left (Figures 6C–E). GOSH plots of the prothrombin time effect size identified four studies, three of which (36, 39, 40) pull effect size to the left (Figures 6F–H), and one (41) pulls effect size to the right (Figure 6I). We have excluded these studies and recalculated the pooled effect size for each parameter. Results are summarized in Table 2.

To explore the possible sources of heterogeneity and test the robustness of our data, we have conducted subgroup analyses based on articles' country of origin and criteria for severity stratification. Results are summarized in Table 3. Subgroup analysis showed no significant difference between articles originated from different countries but revealed that severity assessment criteria might have an influence on overestimated effect sizes for PLT and DD. High heterogeneity levels seem to be persistent oven different subgroups. Being the only exception, values of APTT obtained from non-Chinese articles were less heterogenous–47.1% compared to 84.7% in studies originated in China.

**Table 3:** Summary results of the subgroup analysis.

			**Effect size**		**Heterogeneity**		
**Coag. param**.	**Variable**	**Subgroups**	***k***	**SMD [95% CI]**	***I^**2**^* (%)**	**Tau-Squared**	***p*-subgroup**
PLT	Location						0.2123
		China	19	−0.2256 [−0.3747; −0.0764]	84.60	0.0694	
		Other	15	−0.1384 [−0.3037; 0.0270]	73.60	0.0765	
	Criteria						0.0263
		Guidelines	19	−0.1618 [−0.3319; 0.0082]	85.10	0.0983	
		ICU	9	−0.1188 [−0.3056; 0.0680]	70.60	0.0308	
		Aggravation	3	−0.0948 [−0.8750; 0.6854]	74.60	0.0682	
		Oxygen therapy	3	−0.6834 [−1.4303; 0.0636]	0.00	0	
DD	Location						0.3596
		China	23	0.7618 [0.5089; 1.0148]	94.70	0.3043	
		Other	14	0.5992 [0.3204; 0.8780]	91.60	0.1964	
	Criteria						0.0041
		Guidelines	22	0.6349 [0.4248; 0.8450]	92.10	0.1903	
		ICU	12	0.9257 [0.5007; 1.3506]	96.70	0.4027	
		Aggravation	3	0.2526 [−0.2458; 0.7510]	54.60	0.0182	
FIB	Location						0.0813
		China	11	0.9393 [0.3154; 1.5632]	93.50	0.8093	
		Other	11	0.4162 [0.1757; 0.6567]	74.50	0.0937	
	Criteria						0.1560
		Guidelines	13	0.8539 [0.3128; 1.3949]	92.60	0.7496	
		ICU	8	0.4510 [0.1690; 0.7330]	84.30	0.0814	
		Aggravation	1	0.3031 [0.0253; 0.5809]	–	–	
APTT	Location						0.8248
		China	13	0.2768 [0.0730; 0.4806]	88.30	0.0872	
		Other	8	0.2499 [0.0661; 0.4337]	47.10	0.0169	
	Criteria						0.3590
		Guidelines	16	0.2629 [0.1019; 0.4240]	84.70	0.0636	
		ICU	4	0.3500 [−0.1307; 0.8306]	70.60	0.0520	
		Aggravation	1	0.0714 [−0.2051; 0.3480]	–	–	
PT	Location						0.9249
		China	16	0.2886 [0.1188; 0.4584]	81.80	0.0751	
		Other	8	0.2745 [−0.0244; 0.5734]	78.30	0.1016	
	Criteria						0.6332
		Guidelines	18	0.2889 [0.1401; 0.4377]	79.00	0.0628	
		ICU	5	0.2992 [−0.2853; 0.8836]	87.60	0.1962	
		Aggravation	1	0.1407 [−0.1360; 0.4175]	–	–	

#### Publication Bias

**Figure 7:**
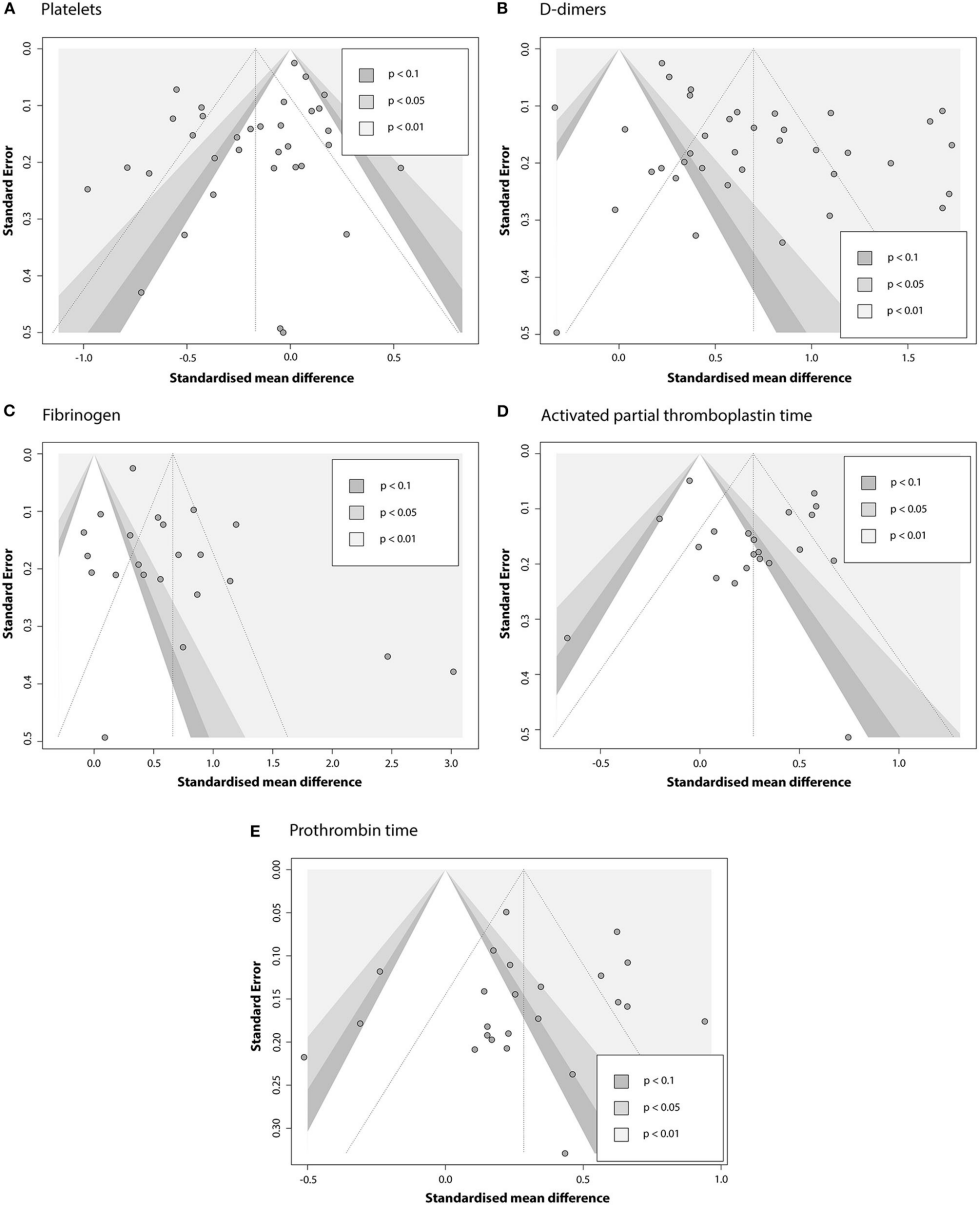
Contour-enhanced funnel plots evaluating the presence of publication bias in the pool of articles that report association between COVID-19 severity and **(A)** PLT; **(B)** DD; **(C)** FIB; **(D)** APTT; **(E)** PT.

Contour-enhanced funnel plots for five coagulation parameters are depicted in Figure 7, and the results of Egger's test are summarized in Table 4. Based on the linear regression results, significant asymmetry is observed in published studies that included results for PLT, DD [Intercept (*p*-value): −1.297 (0.0336), 3.182 (0.0013)], and somewhat significant asymmetry was indicated for FIB [1.872 (0.0493)]. While it is impossible to predict whether asymmetry is coming solely from publication bias, contour-enhanced funnel plots for DD and FIB (Figures 6B,C) show that the number of papers with insignificant findings is very small (*p* > 0.1). This may advocate for the presence of some publication bias. In contrast, Figure 6A illustrates that even though the funnel plot for PLT has some asymmetry, there are plenty of publications with insignificant SMDs, thus decreasing the possibility of publication bias.

**Table 4:** Egger's test for publication bias.

**Parameter**	**Intercept**	**Confidence interval**	***t***	***p*-value**
PLT	−1.297	[−2.44, −0.15]	−2.221	0.0336
DD	3.182	[1.39, 4.97]	3.489	0.0013
FIB	1.872	[0.12, 3.63]	2.093	0.0493
APTT	0.775	[−1.37, 2.92]	0.708	0.4874
PT	−0.436	[−2.49, 1.62]	−0.417	0.6811

To further explore the robustness of our findings, we have conducted several additional tests. As reported by Zwetsloot et al. (42), plotting SMD against its standard error to assess the publication bias can result in distorted funnel plots, leading to false-positive conclusions. Since SMD is necessary for our analysis and cannot be substituted, we used a sample size-based precision estimate instead of the standard error–1/√n. The resulting funnel plots (Supplementary Figure 16) showed no significant distortion.

Similar corrections were made to Egger's test: since standardized mean difference and standard error are not independent, we can expect the test to yield some false-positive results. In our calculations, we used the corrected SE suggested by Pustejovsky and Rodgers (43). Results are given in the Supplementary Table 3. The only model that was affected by this correction was FIB (intercept 2.142 with a *p*-value of 0.0526 compared to the initial 1.872 and 0.0493), indicating that previously concluded asymmetry might have been a false-positive. Interpretation of results for other parameters remained the same.

## Discussion

There is a need to identify early indicators of the risk of critical patients progressing with COVID-19 to ICU. Abnormal coagulation parameters, including DD and FIB levels, and changes in PLT and PT, are observed in many patients with COVID-19 infection at admission [rev. (8, 13)]. However, as discussed by different authors, these indicators provide contradictory information regarding risk stratification and prognosis (44–46). Several previous meta-analysis reviews are researching abnormal coagulation and the severity of COVID-19. The majority of analyzed studies were conducted at the beginning of the pandemic and examined the Chinese population of patients [before April 2020—(14, 47–49); June 2020—(50)]. Our meta-analysis study found that DD and FIB levels, APTT, and prothrombin time were significantly higher in the severe group of COVID-19 patients. In contrast, PLT was significantly lower in these patients. There was no evidence of effects across different geographic subgroups, except for APTT values.

### Platelet Changes

In this meta-analysis, we systematically analyze evidence for the utility of lowered PLT levels on admission in relation to the severity of infection. Platelets play a crucial role in the maintenance of hemostasis, and contribute to thromboinflammatory processes (51). At different stages of viral infection, changes in platelet production or destruction may result in coagulation imbalances leading to pro-thrombotic events or in platelet dysfunction and bleeding risks (52) and increased activation of platelets (19). Thrombocytopenia (platelet count <150 × 10^9^/L) seems to represent an important marker of COVID-19 severity (8, 14, 18, 53) and mortality outcome (14, 18). The reasons for thrombocytopenia are multi-factorial may include early suppression of platelets progenitors' production and damage in bone marrow and lungs, hemophagocytosis and other reasons. Significant thrombocytopenia in COVID-19 is less frequent than it was reported for the largest SARS-1 cohort (45%) (54). SARS-1 induced platelet depletion was related to direct viral infection of megakaryocytes and hematopoietic progenitors [rev. by (4)]. However, almost half of patients developed thrombocytosis following initial thrombocytopenia during the SARS epidemics in 2003 (55). It is possible that opposite PLT changes may reflect different phases of COVID-19 infection (56)—acute and convalescent phases, though the explanation has not been established yet. The reactive thrombocytosis that may occur during the convalescent phase requires time observation, including a period preceding patient discharge. The increase of immature reticulated platelets reported recently in COVID-19 patients by different groups (57, 58) may play a role in the limiting effects of anti-platelet therapies (59–61). Change in platelets parameters (increased mean volume) (62) and reactivity (63, 64) are suggested to be associated with a severe COVID-19 infection (65). In addition to platelet changes, there are an increasing number of studies related to the dysfunction of megakaryocytes (platelets progenitors) in COVID-19 patients. Thus, elevated and abnormal megakaryocytes (MKs) were found in post-mortem and autopsy studies in the lungs (66–69), heart (68), brain (70, 71), bone marrow (72, 73). Recently, the concept that megakaryopoiesis occurs in the lung parenchyma with significant contribution of the lungs in platelet biogenesis has emerged (74–76). There is a close association between platelet biogenesis and alveolarization of the lung (77, 78), and hypoxia-induced thrombocytopenia associated with reduction of lung megakaryocytes and impaired generation of platelets in the lungs (79). The SARS-CoV-2 virus elicited up-regulation of megakaryocyte progenitors and elevated circulating megakaryocytes in blood in severe COVID-19 (80, 81), hypothetically through infection of early megakaryocyte progenitors. Recently, Fortmann et al. (80) detected infected calprotectin+ spike+ megakaryocytes in the blood of patients with severe COVID-19. We hypothesize that viral RNA next may be transferred from megakaryocytes to platelets and circulating plasma EVs. The strong supporting argument is a recent finding of Brunet-Ratnasingam et al. (82) that plasma SARS-CoV-2 RNA is strongly associated with increased mortality. There is a current gap of knowledge regarding platelet non-canonical function and regeneration, and further research is crucial for our understanding of COVID-19 pathogenesis and stratification of increased thromboembolic risk.

### DD and FIB Levels and Thromboembolic Complications

Concordant with other studies, we found DD and FIB levels significantly elevated in severe COVID-19 infection. However, DD levels results may suffer from some publication bias as revealed by meta-regression analysis. It may be related to the variability of cut-offs for varying DD measurement assays used in the different studies [(83); rev. (84)]. The usefulness of hemostasis tests, particularly, D-dimers, is limited by interlaboratory variability reflecting differences in methodologies, antibody sources and specificity, different detection methods, calibrators, and diagnostic thresholds (85, 86). As confirmed by autopsy results, the elevated DD levels can be associated with fibrin deposits within pulmonary extravascular space and alveoli (67), but they may be non-specific to intravascular fibrin formation (87). High DD levels, together with elevated neutrophils, were reported to be predictive factors of pulmonary embolism in COVID-19 patients (88). DD and FIB levels are elevated in both COVID-19 and thromboembolism, and therefore as single tests are unspecific and unhelpful in the differentiation of these conditions. In part, it may be explained by the involvement in abnormal coagulation in COVID-19 of different systems, including endothelial cells, complement activation, and hypofibrinolysis resulting in changes undetected by routine tests (39, 88).

A significantly higher amount of neutrophil extracellular traps [and related markers such as cell-free DNA and myeloperoxidase-DNA (MPO-DNA)] was found when compared with non-COVID-19 thrombi (89). Neutrophil extracellular traps (NETs) markers, and cell-free DNA was most closely aligned with D-dimers, with inflammatory markers (C-reactive protein, lactate dehydrogenase) and more specific markers of NETs, MPO-DNA (90–92). The presence and high concentrations of NETs at COVID-19 patients' tracheal aspirates, blood samples (93, 94), and different organs (73) are associated with COVID-19 severity. The formation of NETs in the lungs and bloodstream can be critically associated with thrombosis (95). Platelet activation and endothelial cell damage (96, 97) in COVID-19 patients are also resulting in elevated levels of extracellular vesicles (EVs) (98–100) expressing tissue factor (101, 102) and associated with severity of disease (99, 101, 103).

### PT, APTT, FIB, and Clot Formation Parameters

PT has been included in the International Society on Thrombosis and Hemostasis (ISTH) criteria for diagnosing disseminated intravascular coagulation (DIC) (104), prolonged at COVID-19 with acute distress respiratory syndrome (ARDS) (10) and is significantly elevated in severe COVID-19 patients (105). Progressive prolongation of PT is considered a predictor of fatal outcome.

The first reports from China described a shortened APTT which is considered to be a predictive marker of hypercoagulation. Later studies reported prolonged APTT (45, 106). A prolongation of APTT may indicate a deficiency of clotting factors (II, V, VIII, IX, X, XI, or XII) or the presence of inhibitor (heparin therapy). Another likely explanation is the presence of antiphospholipid antibodies (aPL) or lupus anticoagulant (LA) observed in 45% of COVID-19 patients in the Mulhouse series (107). In Bowles's study (106), most of the patients with prolonged APTT were positive for LA (91%). However, increasing of heparin therapy usage, especially in severe COVID-19 cases, may also lead to prolonged APTT.

Discordant reports exist concerning the fibrinogen levels and severity of COVID-19 disease. There are reports that COVID-19 is associated with high fibrinogen levels (16, 108) and alternative findings (109). Some authors reported that severe COVID-19 infection induces fibrinolysis shutdown (26, 45), which authors consider partly responsible for high DD and FIB levels. However, there are also reports of an increase in plasmin-antiplasmin complexes in severe COVID-19 and findings of mild consumption coagulopathy seen in a proportion of COVID-19 patients (110). Elevated tissue-type plasminogen activator was also found to be increased in the patients with severe COVID-19 (101).

Fibrinogen is a critical molecule that links coagulation, complement system, and inflammation, and at COVID-19, a significant association between FIB levels and interleukin-6 was described (16). Recent post-mortem studies of COVID-19 patients identified microvascular thrombi in multiple organs, including kidney (67), which may lead to increased DD even in the situation of reduced fibrinolysis. Depression of fibrinolysis was reported not only in ICU patients with severe infection and clinical signs of thromboembolism (26) but also in the general unselected ICU cohort (108, 109). Viscoelastic testing ([ROTEM, tissue-type plasminogen activator (tPA) ROTEM, TEG] and evaluation of clot formation by Clot Waveworm analysis have demonstrated a hypercoagulable state, characterized by increased clot stiffness and severely impaired fibrinolysis (17, 108, 111, 112). However, no consistent association between abnormal VET (viscoelastometric testing) pattern at COVID-19 and clinical outcome have been demonstrated [rev. (113)].

Elevated routine coagulation parameters taken at admission may help with the initial differentiation of severe and non-severe COVID-19 patient groups. The hypothetical mechanisms of fibrinolysis shutdown implicate lowering of fibrinolysis factors (plasminogen) and elevated amounts of inhibitors of fibrinolysis (such as α2-antiplasmin and plasminogen activator inhibitor PAI-1).

Longitudinal observational studies of COVID-19 infection are particularly important for understanding infection dynamics and disease outcomes (114–119). Hardy et al. (117) reported daily changes of relevant parameters of hemostasis of severe COVID-19 patients, including standard hemostatic tests (D-dimers, PT) as well as functional integrative tests for thrombin generation and fibrinolysis. Importantly, the day of hospital and/or ICU admission does not represent the same timepoint in the COVID-19 disease course that increases variability and complicates interpretation of studies results.

Although our findings were consistent across different geographic groups, they were tempered by significant heterogeneity. The DD, FIB, and PLT elevation trends speak about the necessity of investigating the potential of additional specific biomarkers for the stratification of COVID-19 patients. We suggest that thorough characterization of platelets and their progenitors (megakaryocytes), as well as clot formation testing could aid in the assessment of risk forthromboembolic events in COVID-19 patient groups.

## Limitations of the Study

Our findings should be interpreted with some limitations. Firstly, most of the included studies were either retrospective and observational by design, thus prone to recall or misclassification bias. Second, such studies often imply recruitment by convenience sampling, in which case the representativeness of a study is questioned. In the context of this meta-analysis, several different endpoints were allowed: severity guidelines, ICU admissions, oxygen therapy requirement, and disease aggravation. In an attempt to overcome this issue, we have conducted a subgroup analysis. Third, uncontrolled variables might also pose a limitation–both the disease severity and coagulation parameters can be affected by several potential confounding variables, such as comorbidities, age, etc. Fourth, data transformation was applied to those studies that reported their results in quartiles or maximum and minimum values. This could potentially introduce some degree of error in the pooled estimate. Fifth, the random-effects model was used due to the high heterogeneity of research. Although sensitivity and subgroup analyses were conducted, some residual heterogeneity may affect the interpretation of results. Finally, the underlying mechanisms of coagulation disbalances at COVID-19 still need to be investigated.

## Conclusions

Our findings support using a combination of coagulation parameters for risk stratification of patients with COVID-19 infection at the time of admission, in particular DD and FB levels. However, additional evaluation using clotting formation methods or combination with specific biomarkers of production and activation of platelet and platelet progenitors is required. This information may help physicians triage patients with thromboembolism. An important consideration is an intermediate and long follow-up of COVID-19 patients.

## Data Availability Statement

The raw data supporting the conclusions of this article will be made available by the authors, without undue reservation.

## Ethics Statement

The studies involving human participants were reviewed and approved by Ethics Committee of the National Research Center for Cardiac Surgery (NCJSC), Nur-Sultan, Kazakhstan. Written informed consent for participation was not required for this study in accordance with the national legislation and the institutional requirements.

## Author Contributions

PL: conceptualization, methodology, formal analysis, investigation, data curation, and writing original draft. GI, ZS, and AK: methodology, data curation, formal analysis, and investigation. ATT, MS, AD, MA, and MB: methodology, formal analysis, investigation, data curation of NRCSC cohort, and writing a draft. EP and AT: methodology, analysis, and funding. NB: conceptualization, methodology, formal analysis, investigation, data curation, finding, and writing a draft. MB and NB: supervision. All authors read and edit a final draft. All authors contributed to the article and approved the submitted version.

## Funding

NB and AT were supported by FDCRGP SSH2020028 and NB was funded by OPCRP2020018 grants from Nazarbayev University.

## Conflict of Interest

The authors declare that the research was conducted in the absence of any commercial or financial relationships that could be construed as a potential conflict of interest.

## Publisher's Note

All claims expressed in this article are solely those of the authors and do not necessarily represent those of their affiliated organizations, or those of the publisher, the editors and the reviewers. Any product that may be evaluated in this article, or claim that may be made by its manufacturer, is not guaranteed or endorsed by the publisher.

## Supplementary Material

Supplementary Figure 1 Exploring influential cases in meta-analysis models with Baujat plots: **(A)** Platelets, **(B)** D-dimers, **(C)** Fibrinogen, **(D)** Activated partial thromboplastin time, **(E)** Prothrombin time.

Supplementary Figure 2 Influence diagnostic of meta-analysis model for the association of platelet count and COVID-19 severity.

Supplementary Figure 3 Influence diagnostic of meta-analysis model for the association of D-dimers concentration and COVID-19 severity.

Supplementary Figure 4 Influence diagnostic of meta-analysis model for the association of fibrinogen and COVID-19 severity.

Supplementary Figure 5 Influence diagnostic of meta-analysis model for the association of activated partial thromboplastin time and COVID-19 severity.

Supplementary Figure 6 Influence diagnostic of meta-analysis model for the association of prothrombin time and COVID-19 severity.

Supplementary Figure 7 Search for influential cases by leave-one-out method in meta-analysis models for the association of **(A)** platelets count, **(B)** D-dimers and COVID-19 severity.

Supplementary Figure 8 Search for influential cases by leave-one-out method in meta-analysis models for the association of **(A)** fibrinogen, **(B)** APTT and COVID-19 severity.

Supplementary Figure 9 Search for influential cases by leave-one-out method in meta-analysis model for the association of prothrombin time and COVID-19 severity.

Supplementary Figure 10 Forest plots of association of COVID-19 severity and **(A)** platelet counts and **(B)** D-dimers with outliers removed.

Supplementary Figure 11 Forest plots of association of COVID-19 severity and **(A)** fibrinogen, **(B)** activated partial thromboplastin time, and **(C)** prothrombin time with outliers removed.

Supplementary Figure 12 Exploring the influence of meta-analysis study composition on heterogeneity and pooled effect size: **(A)** fibrinogen, **(B)** activated partial thromboplastin time, and **(C)** prothrombin time.

Supplementary Figure 13 Results of three cauterization methods **(A)** K-means, **(B)** DBSCAN, and **(C)** Gaussian Mixture on the meta-analysis model for association of fibrinogen and COVID-19 severity.

Supplementary Figure 14 Results of three cauterization methods **(A)** K-means, **(B)** DBSCAN, and **(C)** Gaussian Mixture on the meta-analysis model for association of activated partial thromboplastin time and COVID-19 severity.

Supplementary Figure 15 Results of three cauterization methods **(A)** K-means, **(B)** DBSCAN, and **(C)** Gaussian Mixture on the meta-analysis model for association of prothrombin time and COVID-19 severity.

Supplementary Figure 16 Exploring presence of publication bias with corrected funnel plots: SMD plotted against sample size-based precision estimate: **(A)** platelet counts, **(B)** D-dimers, **(C)** fibrinogen, **(D)** activated partial thromboplastin time, **(E)** prothrombin time.

Supplementary Table 1 Results of the NIH Quality Assessment.

Supplementary Table 2 Composition of two groups stratified by severity.

Supplementary Table 3 Results of the Egger's test with Pustejovsky's corrected standard error.

Supplementary Data 1 Criteria for clinical severity of COVID-19 used by the National Research Center for Cardiac Surgery.

## Abbreviations

APTT: activated partial thromboplastin time
CI: confidence interval
DD: D-dimer
DIC: disseminated intravascular coagulation
EVs: extracellular vesicles
FIB: fibrinogen
GOSH: graphical display of study heterogeneity
ICU: intensive care unit
MERS-CoV: Middle East respiratory syndrome coronavirus (MERS-CoV)
MPO-DNA: myeloperoxidase-DNA
NRCSC: National Research Center for Cardiac Surgery
PAI-1: plasminogen activator inhibitor
PM: Paule-Mandel tau-squared estimator
PLT: platelets count
PT: prothrombin time
QP: Q-profile
ROTEM: rotation thromboelastometry
SARS-CoV: severe respiratory acute syndrome coronavirus (SARS-CoV)
SMD: standardized mean difference
TEG: thromboelastography
tPA: tissue-type plasminogen activator
VET: viscoelastometric testing
VTE: venous thromboembolism
VWF: von Willebrand factor.
