# Supplementary material for: Meta-Analysis and Systematic Review of Coagulation Disbalances in COVID-19: 41 Studies and 17,601 Patients
Source: Front Cardiovasc Med. 2022 Mar 11;9:794092. doi: 10.3389/fcvm.2022.794092 (PMC8962835; doi:10.3389/fcvm.2022.794092)
Supplement: Supplementary Figure 1 — Exploring influential cases in meta-analysis models with Baujat plots: (A) Platelets, (B) D-dimers, (C) Fibrinogen, (D) Activated partial thromboplastin time, (E) Prothrombin time. [file Data_Sheet_2.docx]

**Meta-analysis of coagulation disbalances in COVID-19: 41 studies and 17601 patients**

**Polina Len^1^, Gaukhar Iskakova^1,#^, Zarina Sautbayeva^1,#^, Aigul Kussanova^1,2,#^, Ainur T. Tauekelova^3^, Madina M. Sugralimova^3^, Anar S. Dautbaeva^3^, Meruert M. Abdieva^3^, Eugene D. Ponomarev^4^, Alexander Tikhonov^1^, Makhabbat S. Bekbossynova^3^, Natasha S. Barteneva^1,6*^**

^1^School of Sciences and Humanities, Nazarbayev University, Kazakhstan

^2^Core Facilities, Nazarbayev University, Nur-Sultan, Kazakhstan

^3^National Research Center for Cardiac Surgery, Nur-Sultan, Kazakhstan

^4^School of Biomedical Sciences, Chinese University of Hong Kong, China

^5^Brigham and Women’s Hospital, Harvard Medical School, Boston, USA

^#^These authors made an equal contribution in the study

Supplementary Material 2


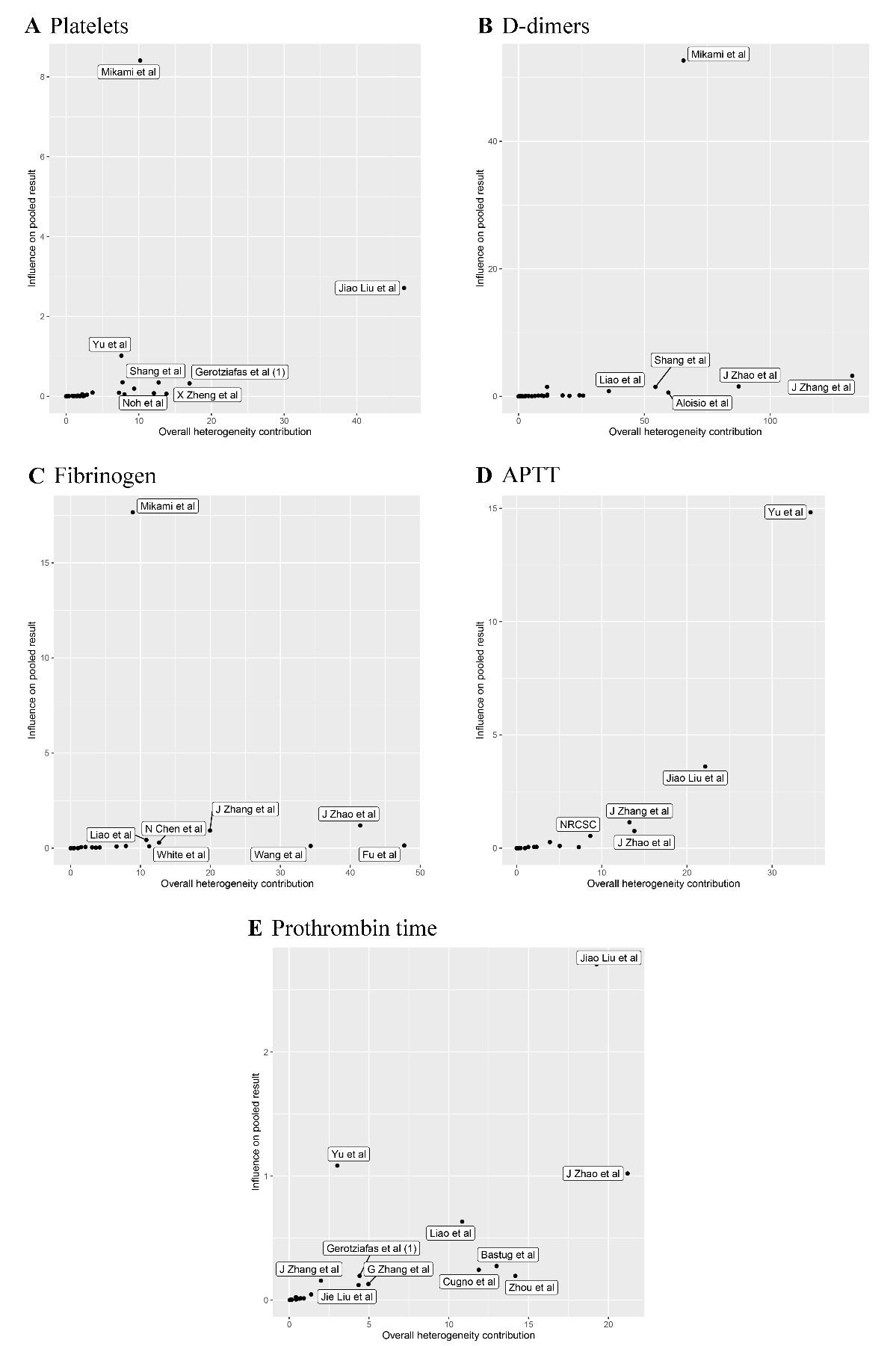


# Supplementary Figure 1. Exploring influential cases in meta-analysis models with Baujat plots: (A) Platelets, (B) D-dimers, (C) Fibrinogen, (D) Activated partial thromboplastin time, (E) Prothrombin time

#
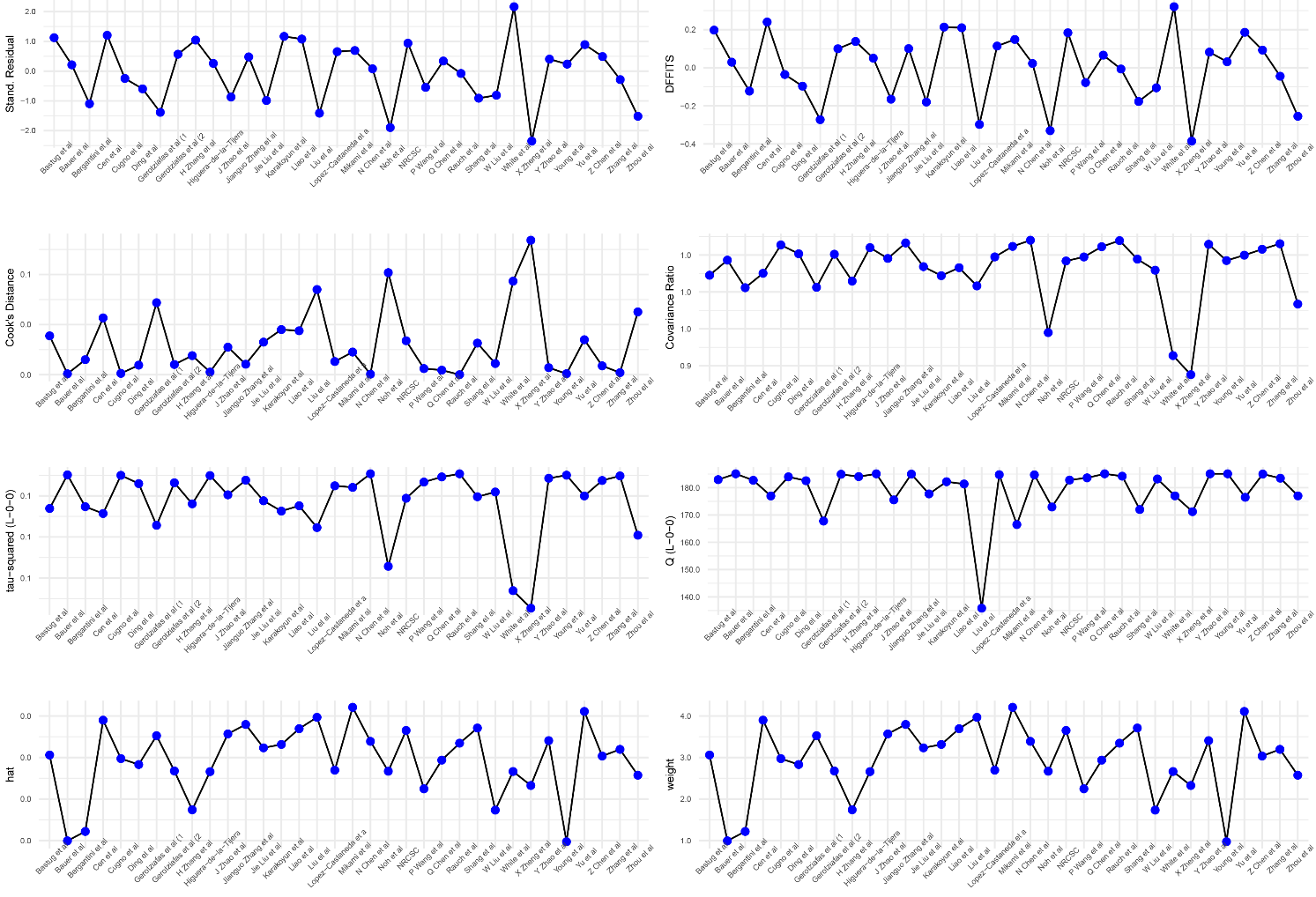


# Supplementary Figure 2. Influence diagnostic of meta-analysis model for the association of platelet count and COVID-19 severity


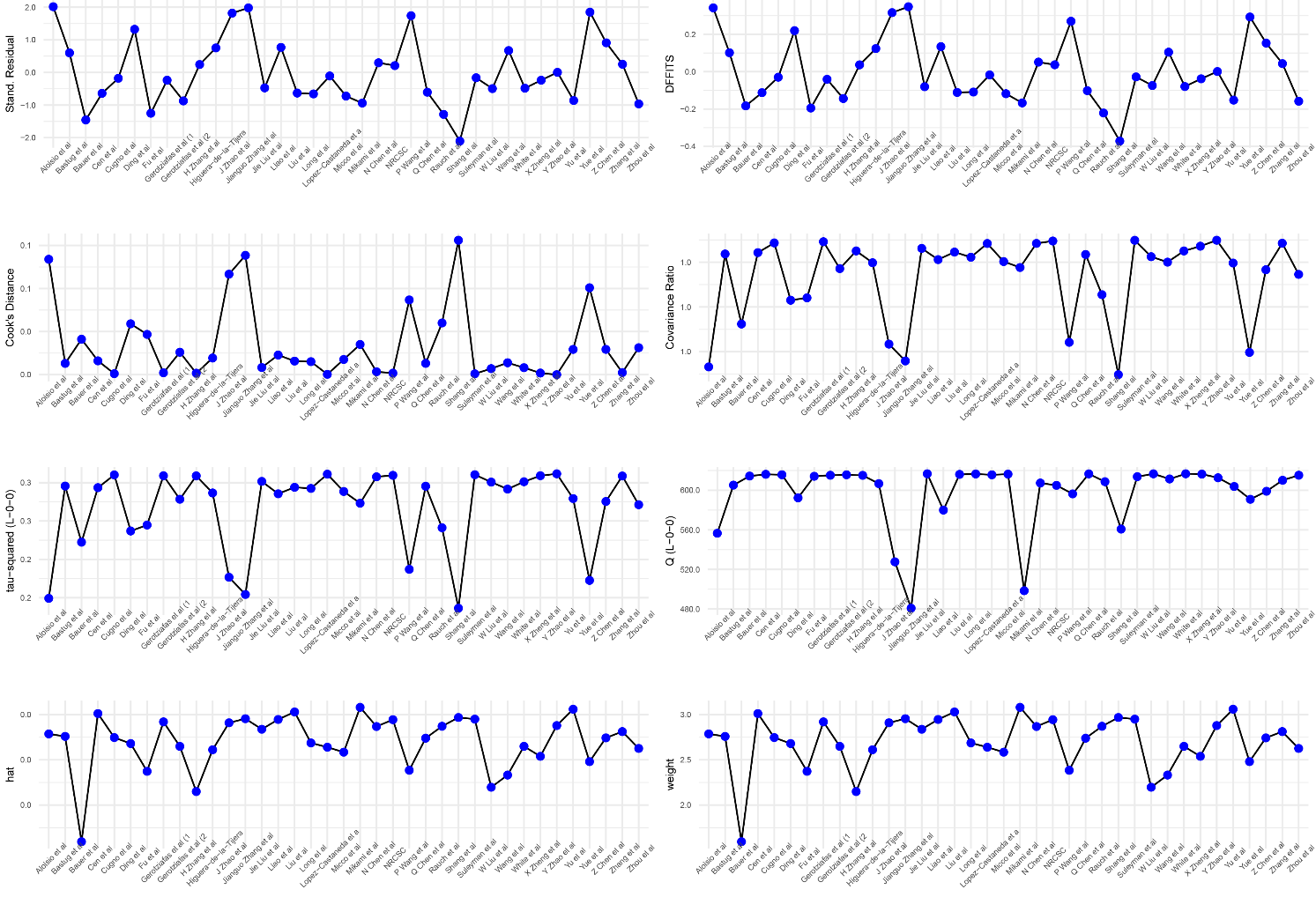


# Supplementary Figure 3. Influence diagnostic of meta-analysis model for the association of D-dimers concentration and COVID-19 severity


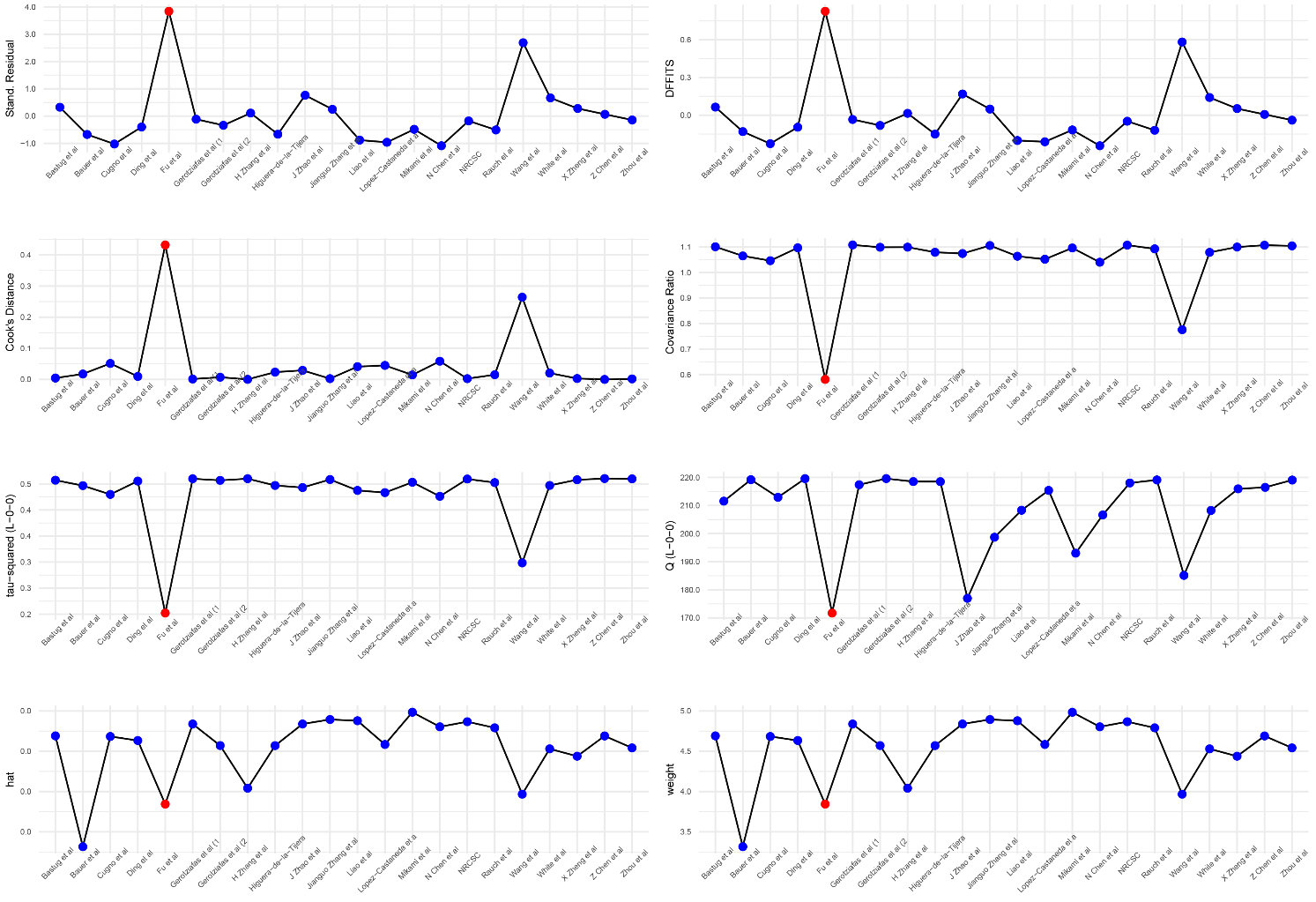


# Supplementary Figure 4. Influence diagnostic of meta-analysis model for the association of fibrinogen and COVID-19 severity


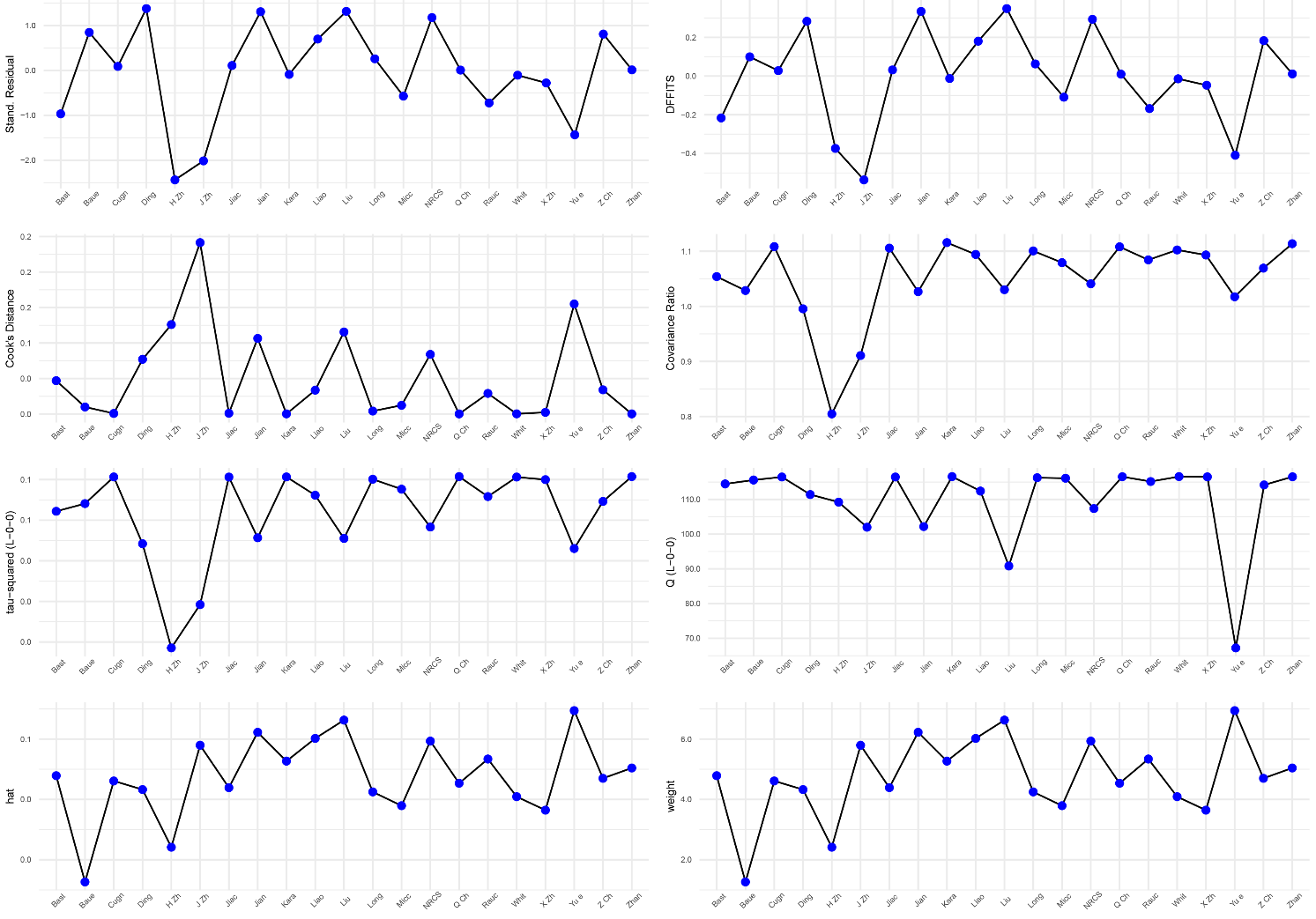


# Supplementary Figure 5. Influence diagnostic of meta-analysis model for the association of activated partial thromboplastin time and COVID-19 severity


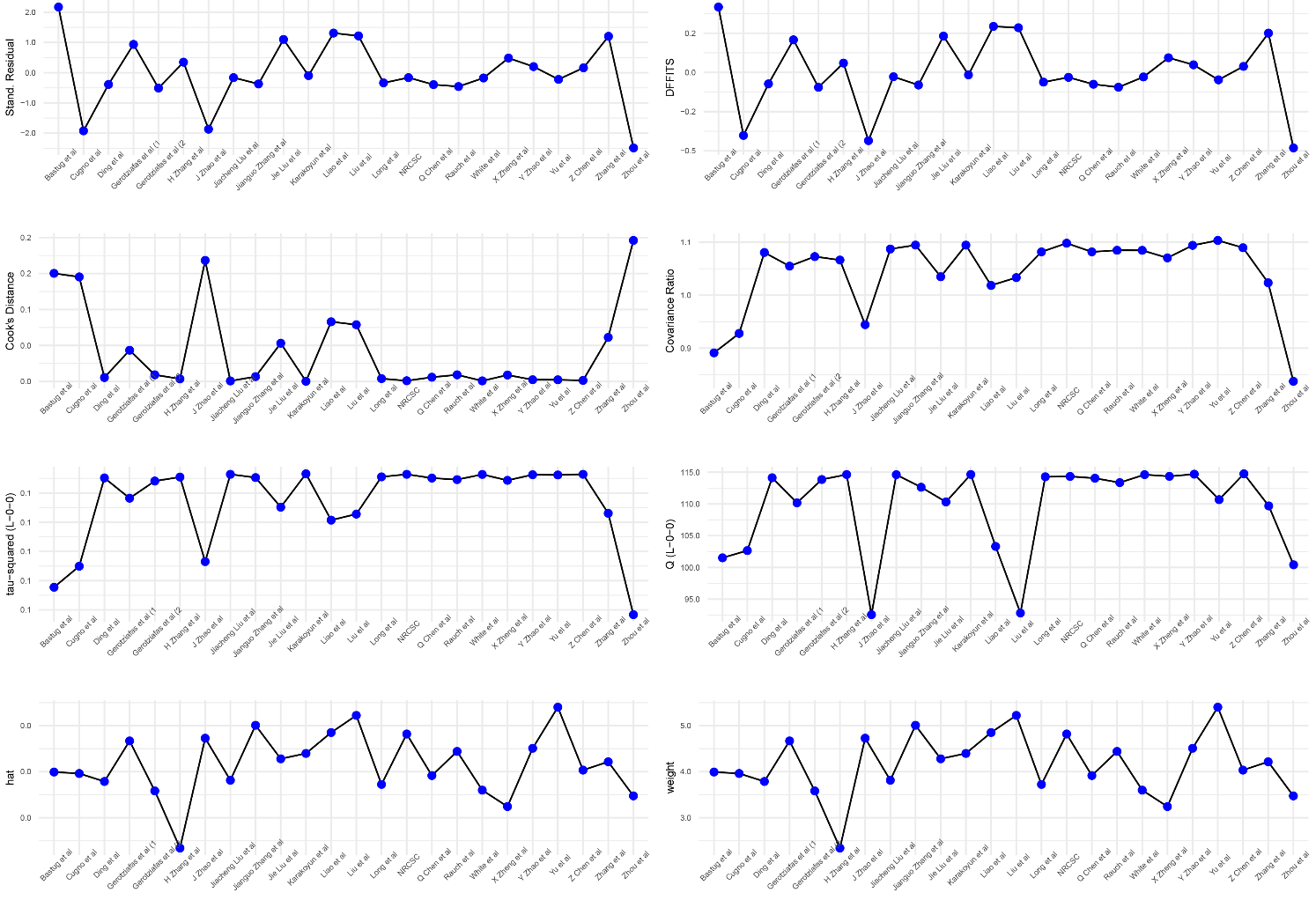


# Supplementary Figure 6. Influence diagnostic of meta-analysis model for the association of prothrombin time and COVID-19 severity


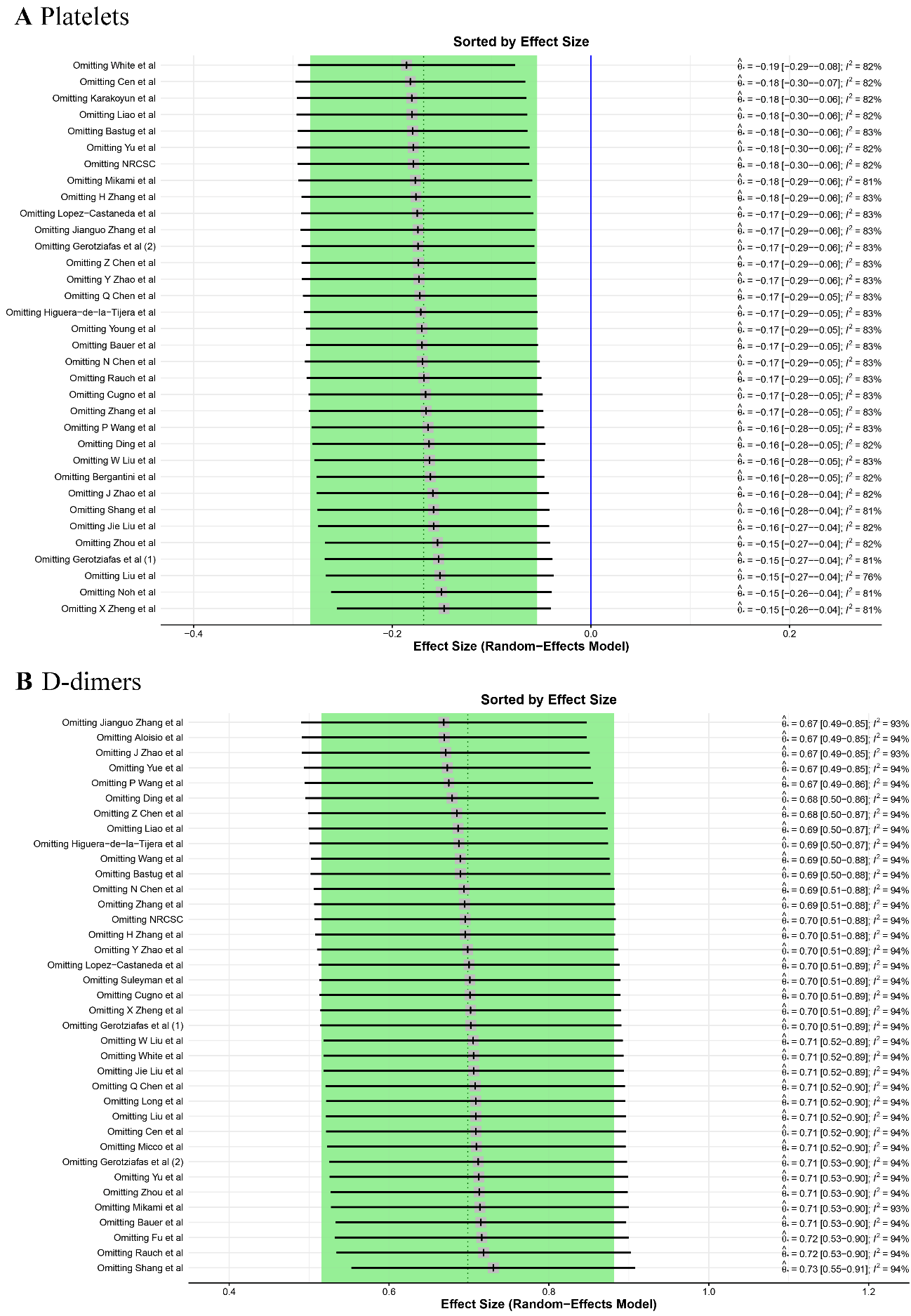


# Supplementary Figure 7. Search for influential cases by leave-one-out method in meta-analysis models for the association of (A) platelet count, (B) D-dimers and COVID-19 severity


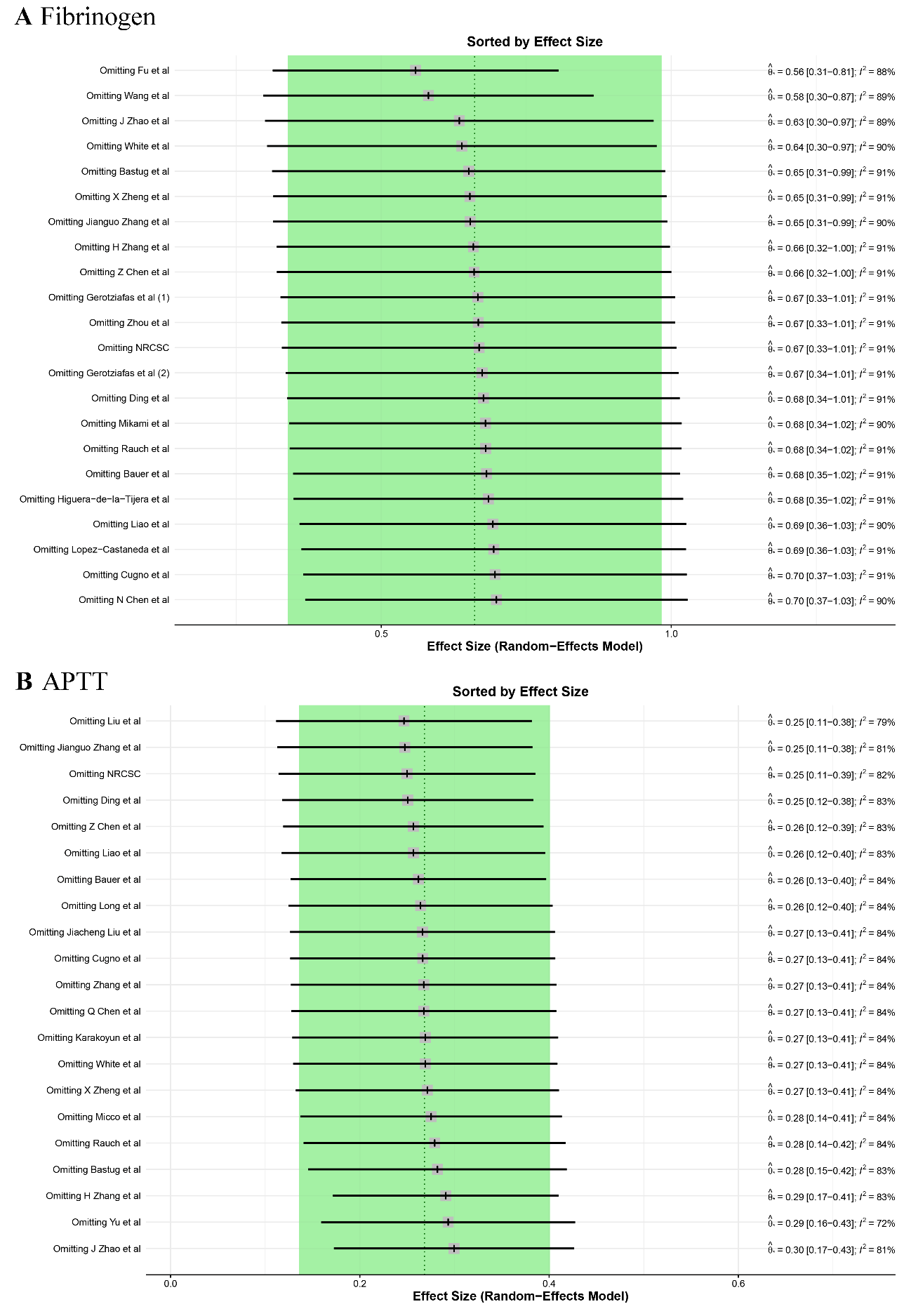


# Supplementary Figure 8. Search for influential cases by leave-one-out method in meta-analysis models for the association of (A) fibrinogen, (B) APTT and COVID-19 severity


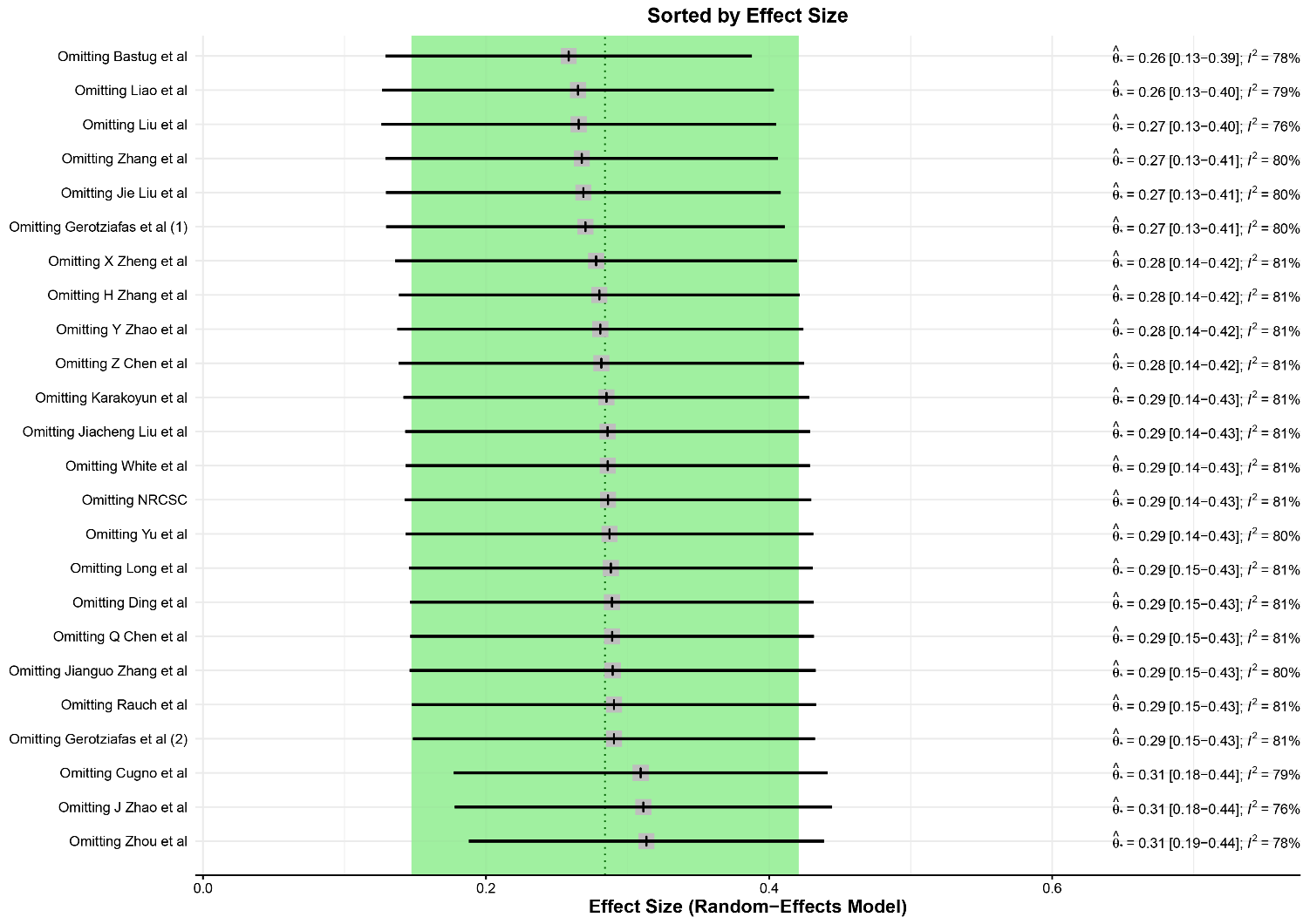


# Supplementary Figure 9. Search for influential cases by leave-one-out method in meta-analysis model for the association of prothrombin time and COVID-19 severity

#
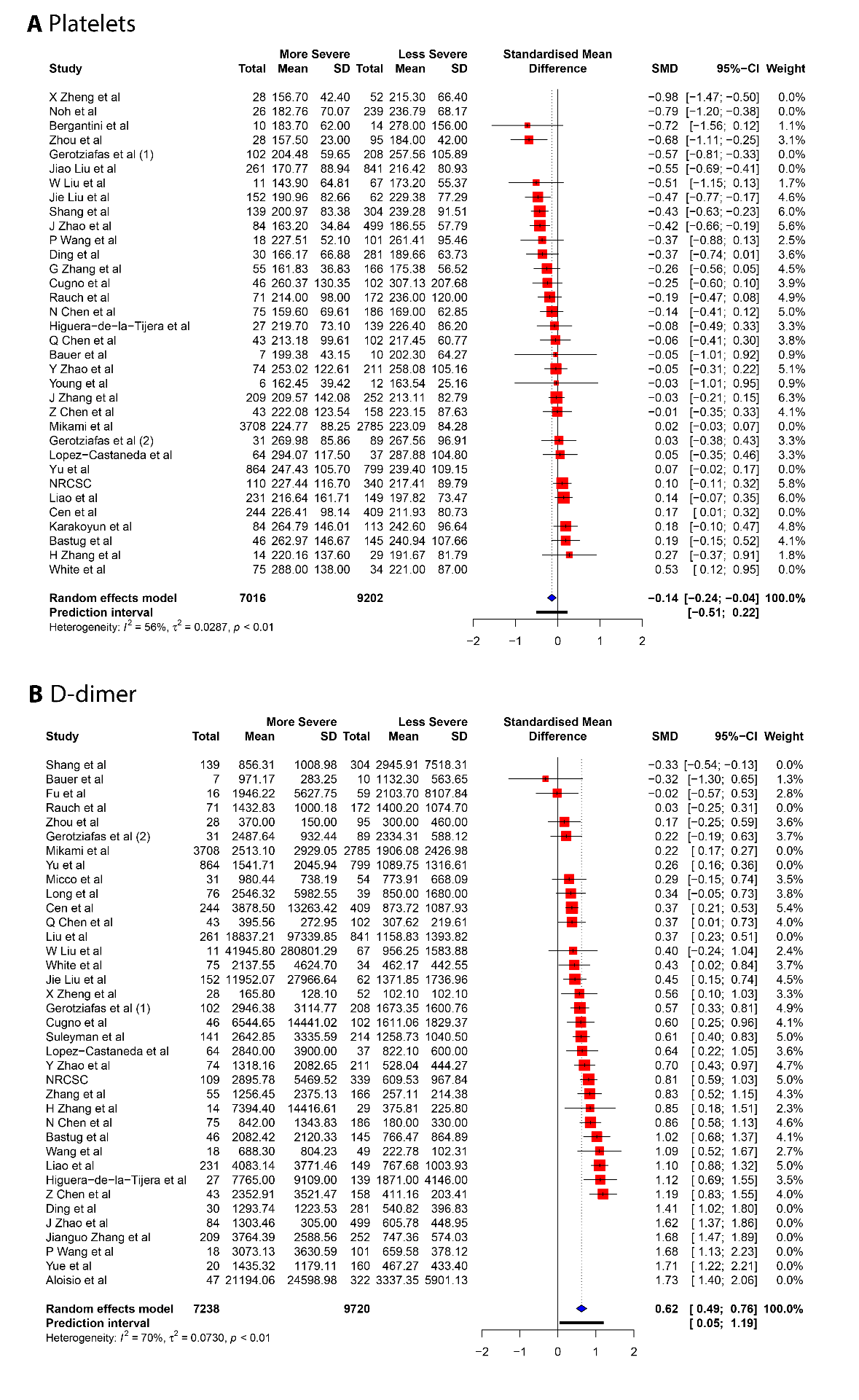


# Supplementary Figure 10. Forest plots of association of COVID-19 severity and (A) platelet count and (B) D-dimers with outliers removed


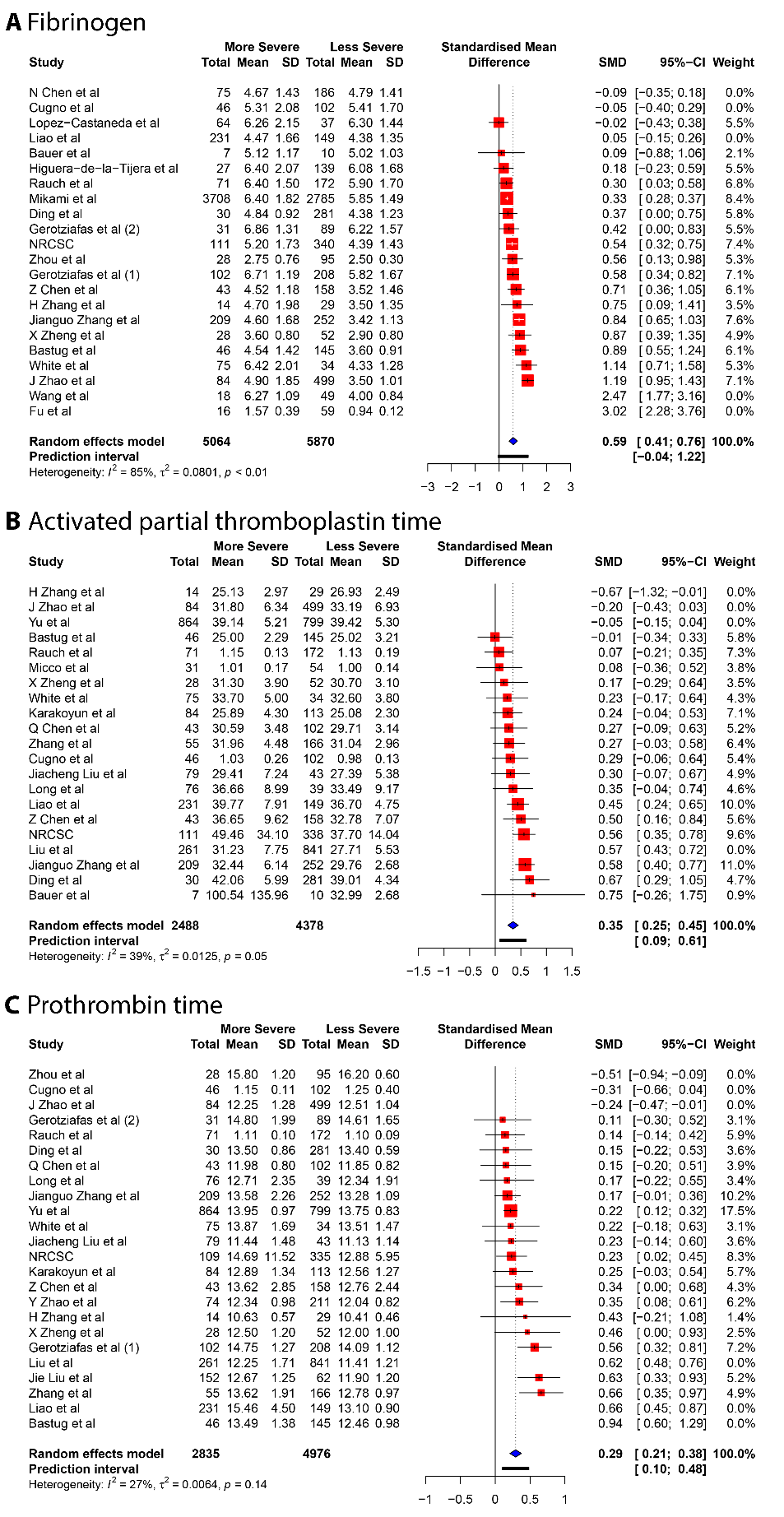


# Supplementary Figure 11. Forest plots of association of COVID-19 severity and (A) fibrinogen, (B) activated partial thromboplastin time, and (C) prothrombin time with outliers removed


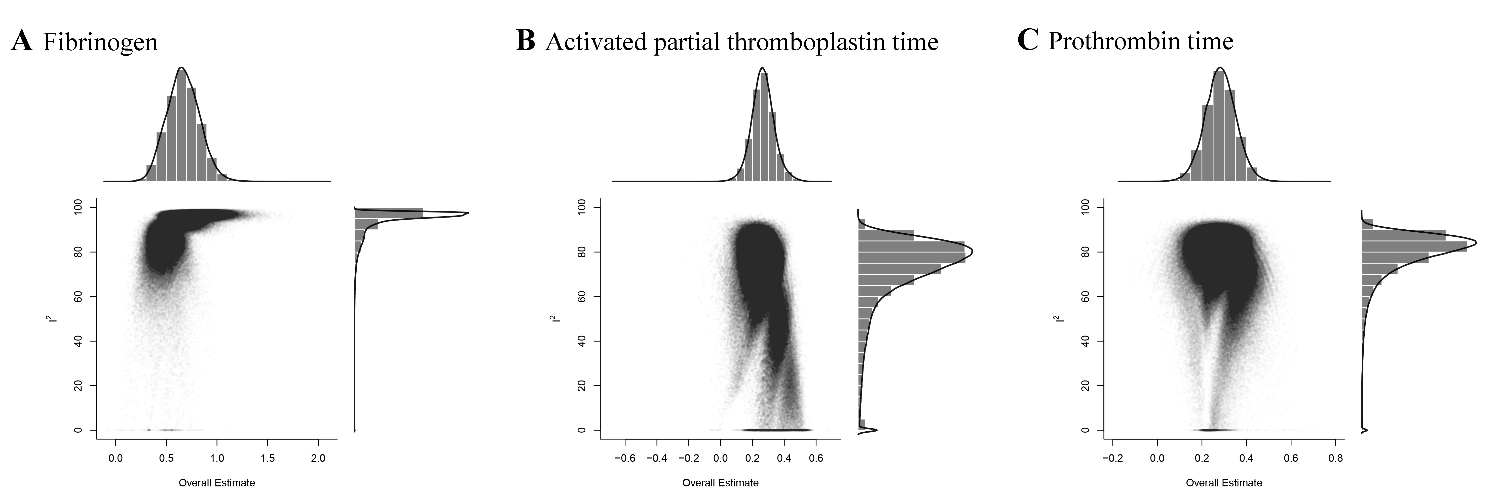


# Supplementary Figure 12. Exploring the influence of meta-analysis study composition on heterogeneity and pooled effect size: (A) fibrinogen, (B) activated partial thromboplastin time, and (C) prothrombin time


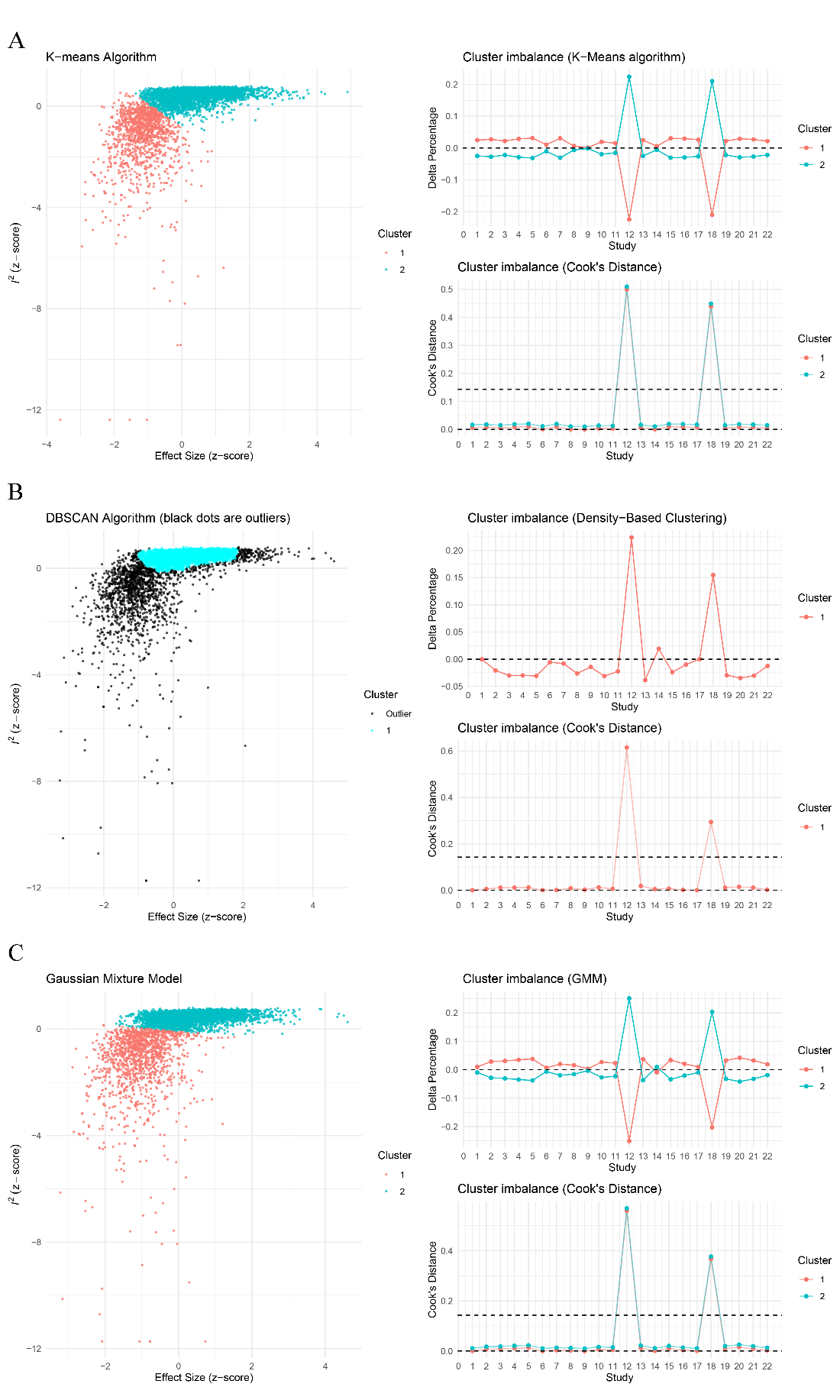


# Supplementary Figure 13. Results of three cauterization methods (A) K-means, (B) DBSCAN, and (C) Gaussian Mixture on the meta-analysis model for association of fibrinogen and COVID-19 severity


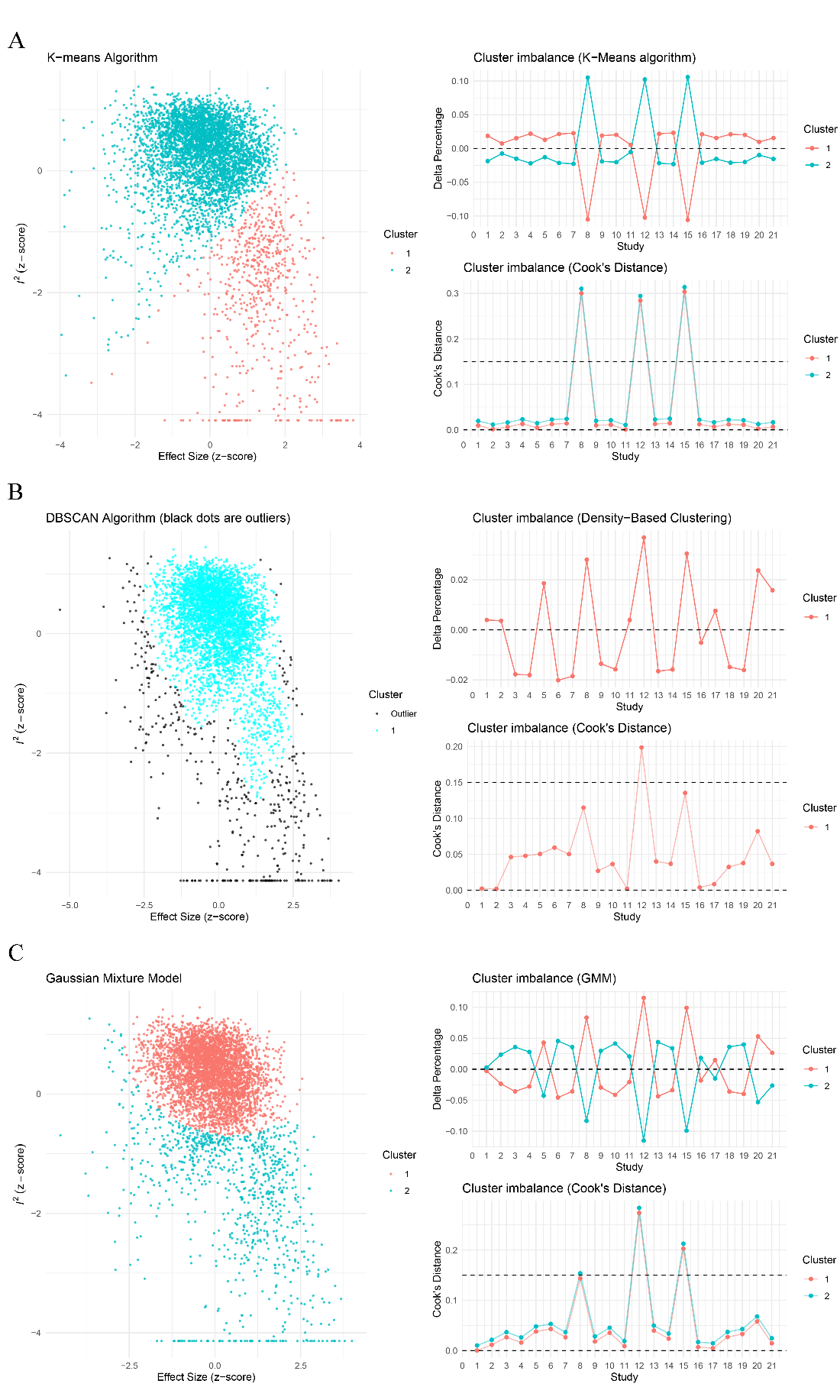


# Supplementary Figure 14. Results of three cauterization methods (A) K-means, (B) DBSCAN, and (C) Gaussian Mixture on the meta-analysis model for association of activated partial thromboplastin time and COVID-19 severity


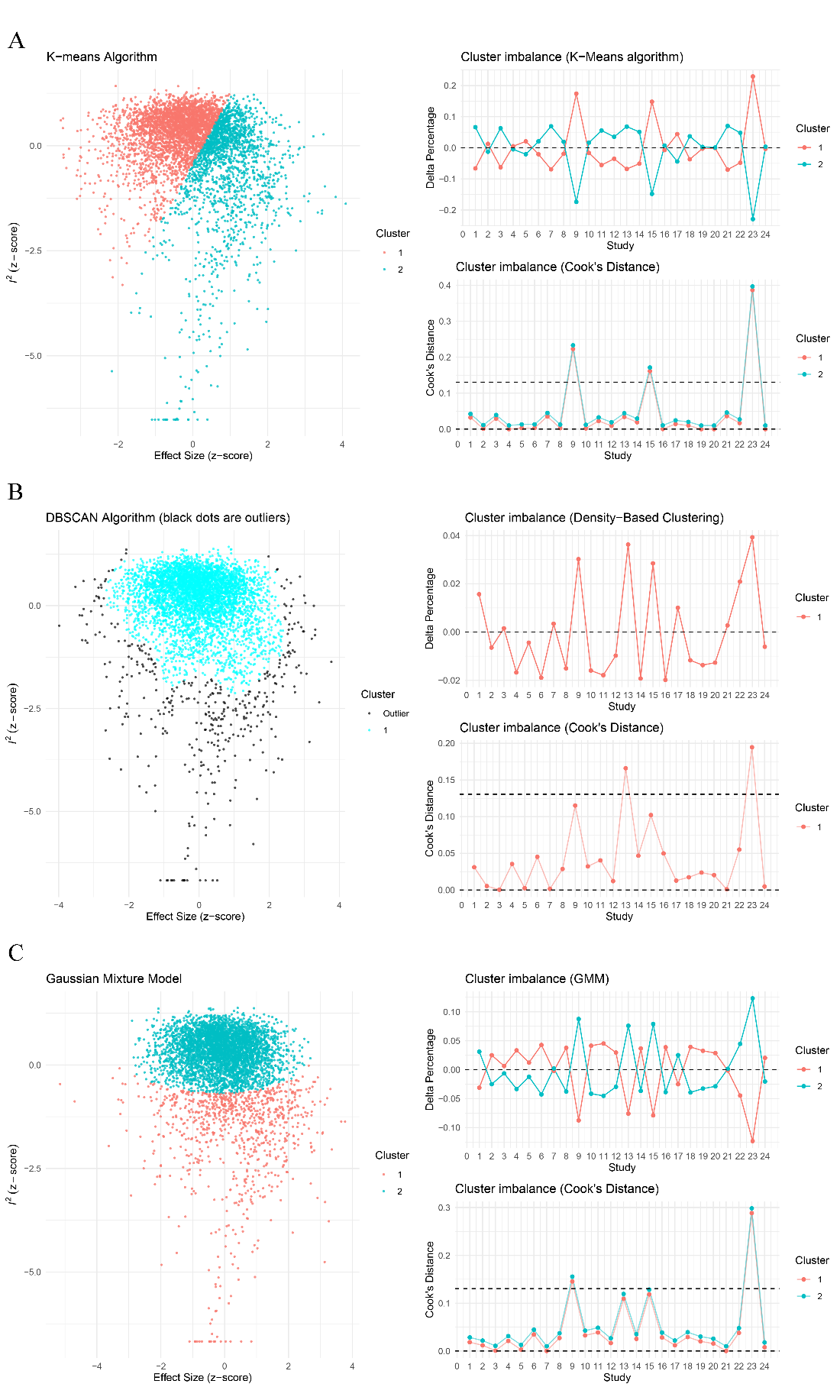


# Supplementary Figure 15. Results of three cauterization methods (A) K-means, (B) DBSCAN, and (C) Gaussian Mixture on the meta-analysis model for association of prothrombin time and COVID-19 severity


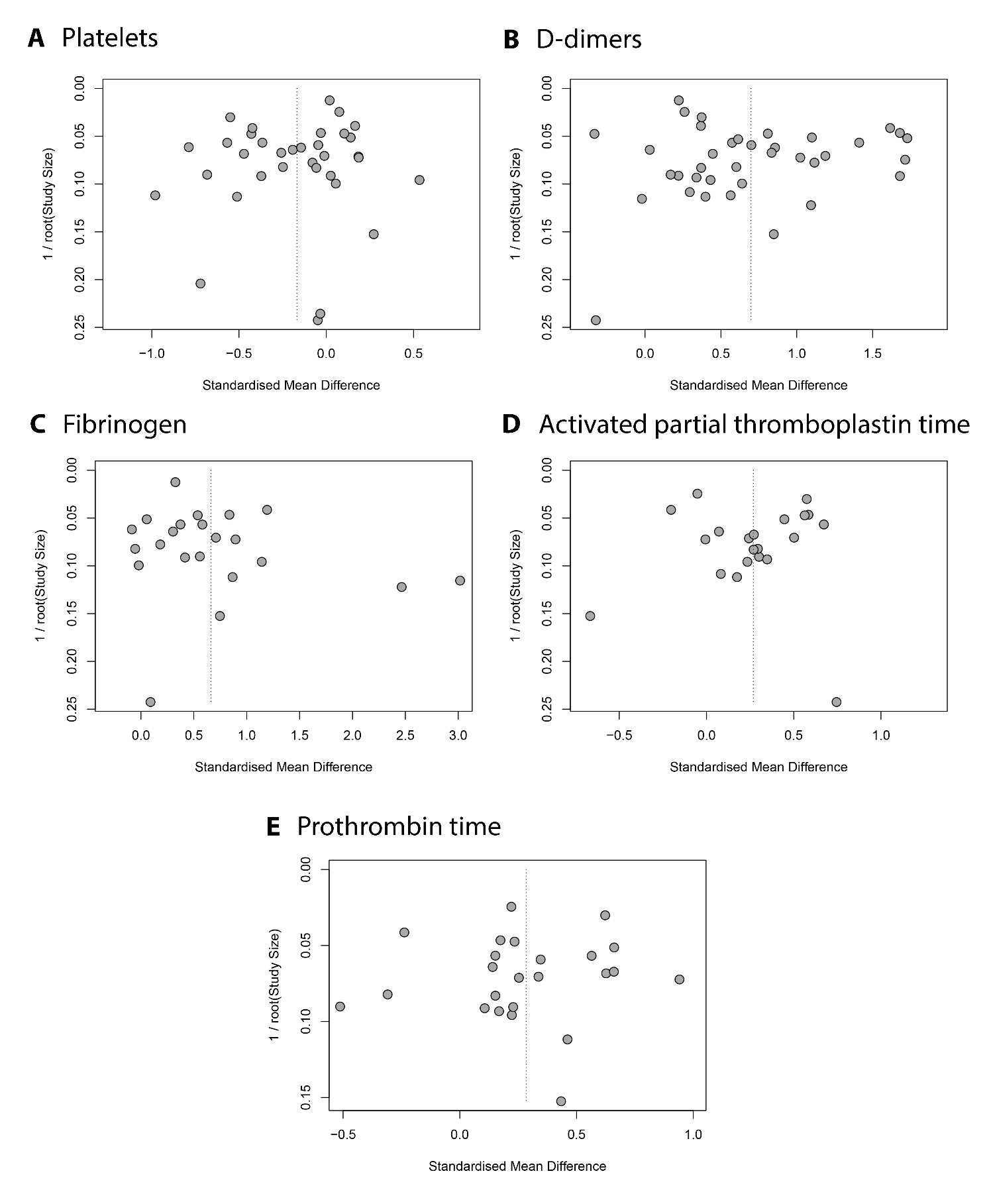


# Supplementary Figure 16. Exploring presence of publication bias with corrected funnel plots: SMD plotted against sample size-based precision estimate: (A) platelet counts, (B) D-dimers, (C) fibrinogen, (D) activated partial thromboplastin time, (E) prothrombin time
