# Supplementary material for: Meta-Analysis and Systematic Review of Coagulation Disbalances in COVID-19: 41 Studies and 17,601 Patients
Source: Front Cardiovasc Med. 2022 Mar 11;9:794092. doi: 10.3389/fcvm.2022.794092 (PMC8962835; doi:10.3389/fcvm.2022.794092)
Supplement: Supplementary Table 1 — Results of the NIH Quality Assessment. [file Data_Sheet_1.docx]

**Meta-analysis of coagulation disbalances in COVID-19: 41 studies and 17601 patients**

**Polina Len^1^, Gaukhar Iskakova^1,#^, Zarina Sautbayeva^1,#^, Aigul Kussanova^1,2,#^, Ainur T. Tauekelova^3^, Madina M. Sugralimova^3^, Anar S. Dautbaeva^3^, Meruert M. Abdieva^3^, Eugene D. Ponomarev^4^, Alexander Tikhonov^1^, Makhabbat S. Bekbossynova^3,*^, Natasha S. Barteneva^1,5,*^**

^1^School of Sciences and Humanities, Nazarbayev University, Nur-Sultan, Kazakhstan

^2^Core Facilities, Nazarbayev University, Nur-Sultan, Kazakhstan

^3^National Research Center for Cardiac Surgery, Nur-Sultan, Kazakhstan

^4^School of Biomedical Sciences, Chinese University of Hong Kong, China

^5^Brigham and Women’s Hospital, Harvard Medical School, Boston, USA

^#^These authors made an equal contribution in the study

Supplementary Data 1

# Supplementary Data 1. Criteria for clinical severity of COVID-19 used by the National Research Center for Cardiac Surgery

- mild - fever <39°С, SpO2 > 95% on room air, no dyspnea, respiratory rate < 20 breaths/min, no imaging findings of pneumonia;
- moderate - fever >38°С, SpO2 94- 95% on room air, dyspnea on exertion, respiratory rate < 20-22 breaths/min, CT extent of lung damage <50%;
- severe – any fever, SpO2 90-93%, dyspnea at less-than-ordinary activity or rest, respiratory rate 23-30 breaths/min, CT extent of lung damage >50%;
- critical - any fever, SpO2 <90%, dyspnea at rest, respiratory rate >30 breaths/min, CT extent of lung damage 75-100%;

# Supplementary Table 1. Results of the NIH Quality Assessment.

|  | 1. Was the research question or objective in this paper clearly stated? | 2. Was the study population clearly specified and defined? | 3. Was the participation rate of eligible persons at least 50%? | 4. Were all the subjects selected or recruited from the same or similar populations ts? | 5. Was a sample size justification, power description, or variance and effect estimates provided? | 6. For the analyses in this paper, were the exposure(s) of interest measured prior to the outcome(s) being measured? | 7. Was the timeframe sufficient so that one could reasonably expect to see an association between exposure and outcome if it existed? | 8. For exposures that can vary in amount or level, did the study examine different levels of the exposure as related to the outcome? | 9. Were the exposure measures (independent variables) clearly defined, valid, reliable, and implemented consistently across all study participants? | 10. Was the exposure(s) assessed more than once over time? | 11. Were the outcome measures (dependent variables) clearly defined, valid, reliable, and implemented consistently across all study participants? | 12. Were the outcome assessors blinded to the exposure status of participants? | 13. Was loss to follow-up after baseline 20% or less? | 14. Were key potential confounding variables measured and adjusted statistically for their impact on the relationship between exposure(s) and outcome(s)? | Score |
| --- | --- | --- | --- | --- | --- | --- | --- | --- | --- | --- | --- | --- | --- | --- | --- |
| Liao et al (1s) | ✓ | ✓ | ✓ | ✓ | ✕ | NA | NA | ✓ | ✓ | NA | ✓ | NA | NA | ✕ | 7 |
| Shang et al (2s) | ✓ | ✓ | ✓ | ✓ | ✕ | NA | NA | ✓ | ✓ | NA | ✓ | NA | NA | ✕ | 7 |
| Rauch et al (3s) | ✓ | ✓ | ✓ | ✓ | ✕ | NA | ✓ | NA | ✓ | ✓ | ✓ | NA | NA | ✕ | 8 |
| Gerotziafas et al  (4s) | ✓ | ✓ | ✓ | ✓ | ✕ | ✓ | ✓ | NA | ✓ | ✓ | ✓ | NA | NA | ✕ | 9 |
| Cen et al (5s) | ✓ | ✓ | NR | ✓ | ✕ | NA | ✓ | ✓ | ✓ | ✓ | ✓ | NA | NA | ✕ | 8 |
| Wang et al (6s) | ✓ | ✓ | ✓ | ✓ | ✕ | NA | NA | ✓ | ✓ | ✓ | ✓ | NA | NA | ✕ | 8 |
| Di Micco et al (7s) | ✓ | ✓ | ✓ | ✓ | ✕ | NA | NA | NA | ✓ | NA | ✓ | NA | NA | ✕ | 6 |
| Bauer et al (8s) | ✓ | ✓ | CD | ✓ | ✕ | NA | ✓ | NA | ✓ | ✓ | ✓ | NA | NA | ✕ | 7 |
| Zhang et al (9s) | ✓ | ✓ | ✓ | ✓ | ✕ | NA | NA | NA | ✓ | NA | ✓ | NA | NA | ✕ | 6 |
| Lopez-Castaneda et al (10s) | ✓ | ✓ | ✓ | ✓ | ✕ | NA | NA | NA | ✓ | NA | ✓ | NA | NA | ✕ | 6 |
| Yue et al (11s) | ✓ | ✓ | ✓ | ✓ | ✕ | NA | NA | NA | ✓ | NA | ✓ | NA | NA | ✕ | 6 |
| White et al (12s) | ✓ | ✓ | NR | ✓ | ✕ | NA | NA | ✓ | ✓ | NA | ✓ | NA | NA | ✕ | 6 |
| Noh et al (13s) | ✓ | ✓ | NA | ✓ | ✓ | NA | ✓ | ✓ | ✓ | ✕ | ✓ | NA | NA | ✓ | 9 |
| Bergantini et al (14s) | ✓ | ✓ | NA | ✓ | ✕ | NA | NA | NA | ✓ | NA | ✓ | NA | NA | ✕ | 5 |
| Liu et al (15s) | ✓ | ✓ | NR | NR | ✕ | NA | ✓ | ✓ | ✓ | ✓ | ✓ | NA | NA | ✓ | 8 |
| Liu et al (16s) | ✓ | ✓ | NR | NR | ✕ | NA | ✓ | ✓ | ✓ | NR | ✓ | NA | NA | ✓ | 7 |
| Zhao et al (36) | ✓ | ✓ | ✓ | ✓ | ✕ | NA | NA | ✓ | ✓ | NR | ✓ | NA | NA | ✓ | 8 |
| Karakoyun et al (17s) | ✓ | ✓ | ✓ | ✓ | ✕ | NA | ✓ | ✓ | ✓ | ✓ | ✓ | NA | NA | ✕ | 9 |
| Zheng et al (18s) | ✓ | ✓ | NA | ✓ | ✕ | NA | ✓ | ✓ | ✓ | ✓ | ✓ | ✓ | NA | ✓ | 10 |
| Zhao et al (40) | ✓ | ✓ | NA | ✓ | ✕ | NA | NA | ✓ | ✓ | NA | ✓ | NA | NA | ✕ | 6 |
| Bastug et al (41) | ✓ | ✓ | NA | ✓ | ✕ | NA | ✓ | NA | ✓ | ✓ | ✓ | NA | NA | ✕ | 7 |
| Liu et al (19s) | ✓ | ✓ | NA | ✓ | ✕ | NA | ✓ | ✓ | ✓ | ✓ | ✓ | NA | NA | ✓ | 9 |
| Fu et al (35) | ✓ | ✓ | NA | ✓ | ✕ | NA | NA | ✓ | ✓ | NA | ✓ | NA | NA | ✕ | 6 |
| H Zhang et al (37) | ✓ | ✓ | NR | NR | ✕ | NA | NA | ✓ | ✓ | NA | ✓ | NA | NA | ✓ | 6 |
| Aloisio et al (20s) | ✓ | ✓ | NA | ✓ | ✕ | ✓ | ✓ | NA | NA | ✓ | ✓ | NA | NA | ✕ | 7 |
| Mikami et al (21s) | ✓ | ✓ | NA | ✓ | ✕ | NA | NA | ✓ | ✓ | NA | ✓ | NA | NA | ✕ | 6 |
| Young et al (22s) | ✓ | ✓ | ✓ | NR | ✕ | NA | NA | NA | ✓ | ✓ | ✓ | NA | NA | ✓ | 7 |
| Higuera-de-la-Tijera et al (23s) | ✓ | ✓ | ✓ | ✓ | ✓ | NA | NA | ✓ | ✓ | NR | ✓ | NA | NA | ✕ | 8 |
| Cugno et al (39) | ✓ | ✓ | CD | ✓ | ✓ | NA | NA | CD | ✓ | NR | ✓ | NA | NA | ✕ | 6 |
| Chen et al (9) | ✓ | ✓ | ✓ | ✓ | ✕ | NA | NA | ✓ | ✓ | ✕ | ✓ | NA | NA | ✓ | 8 |
| Y Wang et al (12) | ✓ | ✓ | ✕ | NR | ✕ | NA | NA | ✓ | ✓ | NR | ✓ | NA | NA | ✕ | 5 |
| Long et al (24s) | ✓ | ✓ | CD | ✓ | ✕ | NA | NA | ✓ | ✓ | NR | ✓ | NA | NA | ✕ | 6 |
| Yu et al (38) | ✓ | ✓ | ✓ | NR | CD | NA | NA | ✓ | ✓ | NR | ✓ | NA | NA | ✓ | 7 |
| Suleyman et al (25s) | ✓ | ✓ | CD | ✓ | ✕ | NA | NA | ✓ | ✓ | NR | ✓ | NA | NA | ✓ | 7 |
| Chen et al (78) | ✓ | ✓ | ✓ | ✓ | ✕ | NA | NA | ✓ | ✓ | NR | ✓ | NA | NA | ✓ | 8 |
| Ding et al (46) | ✓ | ✓ | ✓ | NR | ✕ | NA | NA | ✓ | ✓ | NR | ✓ | NA | NA | ✕ | 6 |
| Chen et al (26s) | ✓ | ✓ | ✓ | ✕ | ✕ | NA | NA | ✓ | ✓ | NR | ✓ | NA | NA | ✕ | 6 |
| Zhang et al (27s) | ✓ | ✓ | ✓ | NR | ✕ | NA | NA | ✓ | ✓ | NR | ✓ | NA | NA | ✕ | 6 |
| Liu et al (28s) | ✓ | ✓ | ✓ | ✓ | ✕ | ✓ | NA | ✓ | CD | ✓ | ✓ | NA | NA | ✓ | 9 |
| Zhou et al (11) | ✓ | ✓ | ✓ | ✓ | ✕ | ✓ | ✓ | ✓ | ✓ | NR | ✓ | NA | NA | ✓ | 10 |

References:

1s. Liao, D., Zhou, F., Luo, L., Xu, M., Wang, H., Xia, J., et al. (2020). Haematological characteristics and risk factors in the classification and prognosis evaluation of COVID-19: a retrospective cohort study. Lancet Haematol. 7, e671-e678. doi: 10.1016/s2352-3026(20)30217-9.

2s. Shang, W., Dong, J., Ren, Y., Tian, M., Li, W., Hu, J., et al. (2020). The value of clinical parameters in predicting the severity of COVID-19. J. Med. Virol. 92, 2188-2192. doi: 10.1002/jmv.26031.

3s. Rauch, A., Labreuche, J., Lassalle, F., Goutay, J., Caplan, M., Charbonnier, L., et al. (2020a). Coagulation biomarkers are independent predictors of increased oxygen requirements in COVID-19. J. Thromb. Haemost. 18, 2942-2953. doi: 10.1111/jth.15067.

4s. Gerotziafas, G. T., Sergentanis, T. N., Voiriot, G., Lassel, L., Papageorgiou, C., Elabbadi, A., et al. (2020). Derivation and validation of a predictive score for disease worsening in patients with COVID-19. Thromb. Haemost. 120, 1680-1690. doi: 10.1055/s-0040-1716544.

5s. Cen, Y., Chen, X., Shen, Y., Zhang, X., Lei, Y., Xu, C., et al. (2020). Risk factors for disease progression in patients with mild to moderate coronavirus disease 2019 - a multi-centre observational study. Clin. Microbiol. Infect. 26, 1242-1247. doi: 10.1016/j.cmi.2020.05.041.

6s. Wang, P., Sha, J., Meng, M., Wang, C., Yao, Q., Zhang, Z., et al. (2020). Risk factors for severe COVID-19 in middle-aged patients without comorbidities: a multicentre retrospective study. J. Transl. Med. 18, 1-12. doi: 10.1186/s12967-020-02655-8.

7s. Di Micco, P., Russo, V., Carannante, N., Imparato, M., Cardillo, G., and Lodigiani, C. (2020). Prognostic value of fibrinogen among COVID-19 patients admitted to an emergency department: an Italian cohort study. J. Clin. Med. 9, 4134. doi: 10.3390/jcm9124134.

8s. Bauer, W., Galtung, N., Neuwinger, N., Kaufner, L., Langer, E., Somasundaram, R., et al. (2021). A matter of caution: coagulation parameters in COVID-19 do not differ from patients with ruled-out SARS-CoV-2 infection in the emergency department. TH Open. 5, e43-e55. doi: 10.1055/s-0040-1722612.

9s. Zhang, J., Huang, X., Ding, D., and Tao, Z. (2021). Platelet-driven coagulopathy in COVID-19 patients: in comparison to seasonal influenza cases. Exp. Hematol. Oncol. 10, 1-13. doi: 10.1186/s40164-021-00228-z.

10s. Lopez-Castaneda, S., García-Larragoiti, N., Cano-Mendez, A., Blancas-Ayala, K., Damian-Vázquez, G., Perez-Medina, A. I., et al. (2021). Inflammatory and prothrombotic biomarkers associated with the severity of COVID-19 infection. Clin. Appl. Thromb. Hemost. 27, 1-9. doi: 10.1177/1076029621999099.

11s. Yue, T., Zhou, W., He, J., Wang, H., Liu, Y., Wang, B., et al. (2021). Combined clinical and imaging features better predict the critical outcomes of patients with SARS-COV-2. Medicine 100, e25083. doi: 10.1097/md.0000000000025083.

12s. White, D., MacDonald, S., Edwards, T., Bridgeman, C., Hayman, M., Sharp, M., et al (2020). Evaluation of COVID-19 coagulopathy: laboratory characterization using thrombin generation and nonconventional haemostasis assays. Int. J. Lab. Hematol. 43, 123-130. doi: 10.1111/ijlh.13329.

13s. Noh, C. S., Kim, W. Y., and Baek, M. S. (2021). Risk factors associated with the need for oxygen therapy in patients with COVID-19. Medicine. 100, e25819. doi: 10.1097/md.0000000000025819.

14s. Bergantini, L., Bargagli, E., d’Alessandro, M., Refini, R., Cameli, P., Galasso, L., et al. (2021). Prognostic bioindicators in severe COVID-19 patients. Cytokine. 141, 155455. doi: 10.1016/j.cyto.2021.155455.

15s. Liu, J., Liu, Z., Jiang, W., Wang, J., Zhu, M., Song, J., et al. (2020a). Clinical predictors of COVID-19 disease progression and death: analysis of 214 hospitalized patients from Wuhan, China. Clin. Res. J. 15, 293-309. doi: 10.1111/crj.13296.

16s. Liu, J., Tu, C., Zhu, M., Wang, J., Yang, C., Liu, W., and Xiong, B. (2021). The Clinical course and prognostic factors of severe COVID-19 in Wuhan, China. Medicine. 100: e23996. doi: 10.1097/md.0000000000023996.

17s. Karakoyun, I., Colak, A., Turken, M., Altin, Z., Arslan, F. D., Iyilikci, V., et al. (2021). Diagnostic utility of C-reactive protein to albumin ratio as an early warning sign in hospitalized severe COVID-19 patients. Int. Immunopharmacol. 91, 107285. doi: 10.1016/j.intimp.2020.107285.

18s. Zheng, X., Chen, J., Deng, L., Fang, Z., Chen, G., Ye, D., et al. (2020). Risk factors for the COVID-19 severity and its correlation with viral shedding: a retrospective cohort study. Int. J. Infect. Dis. 93, 952-961. doi: 10.1002/jmv.26367.

19s. Liu, W., Tao, Z. W., Wang, L., Yuan, M. L., Liu, K., Zhou, L., et al. (2020). Analysis of factors associated with disease outcomes in hospitalized patients with 2019 novel coronavirus disease. Chin. Med. J. 133, 1032–1038. doi: 10.1097/cm9.0000000000000775.

20s. Aloisio, E., Chibireva, M., Serafini, L., Pasqualetti, S.S.F., Dolci, A., Panteghini, M., et al. (2020). A comprehensive appraisal of laboratory biochemistry tests as major predictors of COVID-19 severity. Arch. Pathol. Lab. Med. 144, 1457-1464. doi: 10.5858/arpa.2020-0389-sa.

21s. Mikami, T., Miyashita, H., Yamada, T., Harrington, M., Steinberg, D., Dunn, A., and Siau, E. (2021). Risk factors for mortality in patients with COVID-19 in New York City. J. Gen. Intern. Med. 36, 17-26. doi: 10.1007/s11606-020-05983-z

22s. Young, B. E., Ong, S. W. X., Kalimuddin, S., Low, J. G., Tan, S. Y., Loh, J., et al. (2020). Epidemiologic features and clinical course of patients infected with SARS-CoV-2 in Singapore. JAMA 323, 1488-1494. doi: 10.1001/jama.2020.3204.

23s. Higuera-de-la-Tijera, F., Servín-Caamaño, A., Reyes-Herrera, D., Flores-López, A., Robiou Vivero, E., Martínez-Rivera, F., et al. (2021). The Age-AST-D Dimer (AAD) regression model predicts severe COVID-19 disease. Dis. Markers. 2021,6658270. doi: 10.1155/2021/6658270.

24s. Long, H., Nie, L., Xiang, X., Li, H., Zhang, X., Fu, X., et al. (2020). D-Dimer and prothrombin time are the significant indicators of severe COVID-19 and poor prognosis. Biomed. Res. Int. 2020, 6159720. doi: 10.1155/2020/6159720.

25s. Suleyman, G., Fadel, R. A., Malette, K. M., Hammond, C., Abdulla, H., Entz, A., et al. (2020). Clinical characteristics and morbidity associated with coronavirus disease-2019 in a series of patients in metropolitan Detroit. JAMA Netw. Open. 3, e2012270. doi: 715 10.1001/jamanetworkopen.2020.12270.

26s. Chen, Q., Zheng, Z., Zhang, C., Zhang, X., Wu, H., Wang, J., et al. (2020). Clinical characteristics of 145 patients with coronavirus disease 2019 (COVID-19) in Taizhou, Zhejiang, China. Infection. 48, 543–551. doi: 10.1007/s15010-020-01432-5.

27s. Zhang G, Hu C, Luo L, Fang F, Chen Y, Li J, Peng Z, Pan H. (2020). Clinical features and short-term outcomes of 221 patients with COVID-19 in Wuhan, China. J Clin Virol (2020) 127: 104364. doi: 10.1016/j.jcv.2020.104364

28s. Liu, J., Zhang, S., Wu, Z., Shang, Y., Dong, X., Li, G., et al. (2020b). Clinical outcomes of COVID-19 in Wuhan, China: a large cohort study. Ann. Intensive Care. 10, 1-21. doi: 10.1186/s13613-020-00706-3.

**Supplementary Table 2.** Composition of two groups stratified by severity

| **More Severe** | **Less Severe** |
| --- | --- |
| - Severe | - Mild |
| - Critical | - Moderate |
| - ICU admitted | - Outpatients / ambulatory patients |
| - Oxygen therapy | - No oxygen therapy |
| - Aggravation of disease | - Recovery / Stable |

**Supplementary Table 3**. Results of the Egger’s test with Pustejovsky’s corrected standard error

| Parameter | Intercept | Confidence Interval | t | p-value |
| --- | --- | --- | --- | --- |
| PLT | -1.306 | [-2.47, -0.14] | -2.195 | 0.0355 |
| DD | 3.228 | [1.24, 5.21] | 3.184 | 0.0030 |
| FIB | 2.142 | [0.1, 4.18] | 2.061 | 0.0526 |
| APTT | 0.717 | [-1.46, 2.89] | 0.647 | 0.5255 |
| PT | -0.506 | [-2.59, 1.58] | -0.476 | 0.6389 |
